# Prior home learning environment is associated with adaptation to homeschooling during COVID lockdown

**DOI:** 10.1016/j.heliyon.2022.e09294

**Published:** 2022-04-19

**Authors:** Cléa Girard, Jérôme Prado

**Affiliations:** Lyon Neuroscience Research Center (CRNL), INSERM U1028 - CNRS UMR5292, University of Lyon, Lyon, France

**Keywords:** Covid, Learning inequalities, Home learning environment, Homeschooling, Parental expectation, Parental attitude, Learning time

## Abstract

The COVID-19 crisis in 2020 led to exceptional measures to contain the spread of the virus. In France as in many countries around the world, the government ordered a lockdown with school closure for several weeks. A growing number of studies suggest that family socio-economic status might be an important predictor of how families adapted to homeschooling during lockdown. However, socio-economic status is a distal factor that does not necessarily inform on the specific characteristics of the home learning environment that may more directly influence parental adaptation to homeschooling during lockdown. Here we aimed to examine how parental adaptation to homeschooling during lockdown was influenced by prior parental attitudes and expectations towards academic learning, as well as prior familiarity with literacy and numeracy activities at home. The present study involves 52 families who participated in a study about the home learning environment in 2018. At that time, parents completed an extensive questionnaire assessing their beliefs and attitudes towards academic learning and the frequency of literacy and numeracy activities are home. At the end of the first 2020 French lockdown, we again asked the same parents to complete a questionnaire, this time assessing homeschooling conditions during lockdown as well as parental confidence towards academic domains. Over and above a range of background variables, correlation analyses revealed that parental expectations towards academic learning as well as frequency of prior shared activities were related to daily homeschooling time during lockdown. Both parental attitudes and expectations towards numeracy and literacy were also related to parental confidence in homeschooling. Our results suggest that several aspects of the home learning environment may have influenced how families adapted to homeschooling during the 2020 COVID lockdown.

## 1 Introduction

The COVID-19 pandemic has led to unprecedented consequences in education. Faced with the spread of the virus, the first response of many countries around the world was to reduce physical contact, notably by closing schools and sending children home (Viner et al., 2020). In France, the first lockdown lasted 2 months, from March to May 2020. During that period, all schools closed and children had to be homeschooled (Hamilton et al., 2020). In France, while the government attempted to maintain a continuity of education by providing teachers with online pedagogical tools, teachers largely relied on parents for supervising homework as learning materials were mostly sent to parents by email (Champeaux et al., 2020). As in other countries implementing similar measures, the consequences of school closures for children have quickly become a major concern. For example, the French Pediatric Society recommended a return to normal schooling conditions as soon as possible (Cohen et al., 2020).

Concerns about homeschooling during lockdown grew out of prior research investigating the effects of school interruption on children's learning. This notably involved absenteeism (Santibañez and Guarino, 2021), holidays (von Hippel and Hamrock, 2019) or strikes (Jaume and Willén, 2019). This research suggests that an interruption of formal schooling typically widens socio-economic inequalities in education outcomes (Alexander et al., 2007). Interestingly, these data can be used to project the potential impact of COVID-related lockdowns on learning (Azevedo et al., 2020; Bao et al., 2020; Kuhfeld et al., 2020). For example, Bao et al. predicted an important learning loss due to COVID-related lockdown, but less so for children who have books at home and are used to read daily (Bao et al., 2020). In Europe, it has been suggested that even a "conservative estimate" of the consequences of school closures may translate into a significant learning loss and an exacerbation of existing educational inequalities (Di Pietro et al., 2020). Although the effects of school interruptions on children's learning might be mediated by a general lack of instructional time, it is important to consider that in many cases schools tried to maintain some contact with children during lockdown through online instruction (Bonal and González, 2020). Because some children might have easier access to online education than others, it is possible that the reliance on online learning may have increased inequalities even further (Lancker and Parolin, 2020).

Several studies have already been able to take advantage of current data collected after COVID-related lockdowns to evaluate the consequences on children's learning. For instance, comparing mock exam scores of Chinese university applicants before and after lockdown, Cai et al. (2020) found that a lockdown disproportionately affected economically disadvantaged students. In a large-scale study of Dutch school children, Engzell et al. (2021) found lockdown-related learning losses that were larger in children whose parents were less educated. Similar results were obtained by Maldonado and De Witte (2020) in a study of primary school children in Belgium. Finally, Domingue et al. (2021) examined the effects of school closure on oral reading fluency assessments of children in the US. They found that second and third grades were behind expectations after lockdown, but again with an inequitable impact between advantaged and disadvantaged children.

In France, an examination of homeschooling conditions during the lockdown indicates that teachers relied on online resources for only 20% of activities for primary school children (vs. 60% in other countries such as Italy). In contrast, learning content was mostly sent by email to parents (Champeaux et al., 2020). Therefore, in France (as in other countries), homeschooling relied heavily on parents (especially in the case parents of younger children, Petretto et al., 2020). To truly understand the differential effects of homeschooling during COVID lockdown, it is important to better understand the actual experience of children within the home environment during that period. To date, most reports have explored the time spent working by children, either through parental surveys (Andrew et al., 2020; Bonal and González, 2020; Brom et al., 2020; Champeaux et al., 2020; Domina et al., 2021; Gao et al., 2020; Grewenig et al., 2020) or by combining demographic measures with indirect measures such as data from reading software (Reimer et al., 2021), families' library takeout data of digital children's books (Jæger and Blaabæk, 2020) or google searches on online learning resources (Bacher-Hicks et al., 2021). All of these studies have shown a significant reduction in the time children spent working, as compared to pre-lockdown baseline (Brom et al., 2020; Champeaux et al., 2020; Grewenig et al., 2020). However, parental socio-economic status (SES), an index of family status and position in society, influenced learning time in most studies (i.e., children from disadvantaged backgrounds spent significantly less time working; Andrew et al., 2020; Bacher-Hicks et al., 2021; Bonal and González, 2020; Domina et al., 2021; Jæger and Blaabæk, 2020; Meeter, 2021; Reimer et al., 2021).

Overall, studies support the idea that SES is a major factor influencing the way families have adapted to school closures during lockdown, with children from disadvantaged families being the most affected. However, it is important to consider that SES is a relatively distal construct that may have affected families' responses to school closures through more proximal factors. These might include parental skills, beliefs, and attitudes towards academic learning, but also a pre-existing familiarity with academic activities in the home environment. Indeed, a growing body of research indicates a large variability in the frequency of literacy and numeracy activities that are typically shared between parents and children at home (LeFevre et al., 2009; Sénéchal, 2006). Studies on this so-called “home learning environment” show that such activities, but also parental beliefs and attitudes towards academic learning, are related to children's literacy (Molfese et al., 2016; Sénéchal and LeFevre, 2014; Sénéchal and Young, 2008) and numeracy (Daucourt et al., 2021; Elliott and Bachman, 2018; Mutaf-Yıldız et al., 2020) skills. For example, studies have found relations between children's vocabulary skills and the frequency of shared book reading (Bus et al., 1995; Fletcher and Reese, 2005), or between children's numerical skills and the frequency of board game activities (de Chambrier et al., 2021). It is important to note, however, that there are inconsistencies between studies (Daucourt et al., 2021). Some have proposed that inconsistencies may be due to the fact that the effect of home practices on children's academic skills might be modulated by both the type of practice (e.g., formal versus informal) and its level of complexity (at or above the child's level) (Skwarchuk et al., 2014), though this idea is not always supported by studies (Huntsinger et al., 2016). Recent studies have also highlighted the need to control for several confounding factors when examining the role of the home learning environment, such as parental skills (Hart et al., 2021; van Bergen et al., 2016), children's cognitive factors (Kleemans et al., 2016) or other family variables (Hornburg et al., 2021; Napoli et al., 2021). In any case, with the exception of a few studies investigating parental beliefs and feelings of ability during COVID-related lockdowns (Bol, 2020; Dong et al., 2020; Garbe et al., 2020), it remains unclear to what extent the home learning environment might have affected families' response to school closures.

## 2 The present study

In the present study, we explored how the way parents adapted to homeschooling during lockdown was influenced by prior parental beliefs and attitudes towards academic learning as well as prior familiarity with literacy and numeracy activities at home. At the end of the first 2020 French lockdown (involving school closure), we contacted several families who already came to our lab two years earlier for a study of the home learning environment (Girard et al., 2021a). At that time (T1), both parents and children (who were then 8 years of age) completed tests of reading fluency and arithmetic fluency, while parents also responded to a questionnaire assessing their beliefs and attitudes towards academic learning and the frequency of literacy and numeracy activities are home. At the end of the lockdown (T2), we again asked parents to complete a questionnaire, this time assessing the homeschooling conditions during lockdown as well as parental confidence towards academic subjects. Children also completed an anxiety scale and their reading and arithmetic skills were measured using online tasks.

We hypothesized that the quality of the home learning environment measured before lockdown (as indexed by the frequency of literacy and numeracy practices but also by parental beliefs and expectations towards academic learning) would be associated with differences in adaptation to homeschooling during lockdown. Specifically, a higher-quality home learning environment prior-lockdown might be associated with increased learning time, time spent by parents supervising learning, and parental confidence towards homeschooling during lockdown. Critically, it is possible that these associations might be confounded by academic skills of both parents and children, perceived levels of stress of parents and children, as well as physical living conditions at home during the lockdown period. Therefore, all of these variables were measured in the present study.

## 3 Material and methods

### 3.1 Participants

Participants were *n =* 52 elementary school children (*M =* 10.43 years, range = 9.57–11.29, *n =* 17 females) and one of their parents (*n =* 48 females). Children had no history of neurological disease, mental disorders, or attention deficits, as indicated by their health record. All children and parents were native French speakers. Out of the *n* = 52 children, *n* = 21 were in 4^th^ grade, *n* = 29 were in 5^th^ grade and *n* = 2 were in 6^th^ grade. Children (and parents) came to the lab approximately 2 years before the present study (age of children at that time: *M =* 8.45 years, range = 8.02–09.22) for an evaluation of their skills and of the home learning environment (i.e., T1). At T1, children's full-scale IQ, assessed with the NEMI-2 (Cognet, 2006), was normal to high-normal, ranging from 90 to 135 (*M* = 113, *SD* = 10.5).

All families participating in that study (*n* = 66) were recontacted through email for this follow-up study (i.e., T2). Therefore, there was a 21% attrition rate between T1 and T2. Children in families that did not respond to our request for a follow-up were not different from those in families that responded positively with respect to sex, skills, IQ, home practices, parental income and parental education (all *ps* > .19). The study was approved by the local ethics committee (CPP Sud-Est 2). Families were paid 40 euros for their participation in the first testing session and children were offered a gift for their participation in the second online testing session.

### 3.2 Measures at T1 (two years before lockdown)

At T1, both parents and children came to the lab for an extensive testing session. This was done in the context of a study on the home learning environment (more information about that study can be found in Girard et al., 2021a; 2021b, 2022). Parents were given an electronic questionnaire on a tablet. The questionnaire first assessed family SES based on parents' education level and monthly income. Since it has been recommended not to combine these variables to avoid confounding their potentially distinct influences (Bradley and Corwyn, 2002; Braveman et al., 2005; O'Connell, 2019), they were considered as two separate indicators of SES in our study.

Education level was measured using the number of years pre- or post-high school graduation, with high school graduation set at 0 (i.e., positive numbers indicated that parents completed some years of education after graduating). Approximate monthly income was measured using ranges with increments of €1,000. The minimum possible range was €0 to €999 and the maximum possible range was more than €10,000. For all analyses, we used the median of the reported income range (e.g., €500 for a range between €0 to €1,000) as an approximation of parents’ income. At T1, parental income ranged from less than €12,000 to more than €60,000 per year (*M* = 20,724 €). Ten percent of parents reported to only have a secondary degree, 58% reported having an undergraduate degree, and 32% a master degree or higher. Socio-economic status remained stable for a large majority of parents between T1 and T2. Ten percent of them declared a change in their level of monthly income (among them, 6% declared an average increase of 1,000 euros per month whereas 4% declared an average decrease of 1,000 euros per month). Therefore, SES ranged from low to high at both T1 and T2.

The questionnaire then assessed parental attitudes and expectations towards numeracy and literacy, as well as the frequency of home numeracy and literacy activities. Items were adapted and translated from a questionnaire developed by LeFevre et al. (2009) and are presented (in English translated from original French) with their descriptive statistics in Table S1. Briefly, parents were first asked to describe on a five-point rating scale (i.e., Not sure, strongly disagree, disagree, Agree, Strongly agree) their past and current attitudes toward numeracy (6 items, scale ranges = -1.5–1.5, *M* = 0.64, Crohnbach's alpha = 0.68) or literacy (6 items, scale ranges = -1.5–1.5, *M* = 0.98, Crohnbach's alpha = 0.55) (e.g., “I find math/reading enjoyable” or “When I was at school, I was good at math”; for a complete list of items see S1 Table). Parental expectations regarding numeracy (13 items, scale ranges = -3 **-** 3, *M* = 2.07, Crohnbach's alpha = 0.90) and literacy (12 items, scale ranges = -3 **-** 3, *M* = 2.01, Crohnbach's alpha = 0.89) learning for their child were explored by asking parents to rate on a six-point rating scale how important it is for them that their child acquires a given skill by the end of elementary school (i.e., no opinion, really not important, not important, important, very important, extremely important). For home learning activities, parents were asked how often they engaged in home learning activities that involved numeracy (36 items, scale ranges = 0–5, *M* = 1.48, Crohnbach's alpha = 0.90) or literacy with their child (18 items, scale ranges = 0–5, *M* = 2.07, Crohnbach's alpha = 0.74; for a list of activities and their descriptive statistics, see S1 Table).

Finally, math and reading skills of both parents and children were assessed using the same math fluency (Math Fluency subtest of the Woodcock-Johnson Test of achievement; WJ III Woodcock et al., 2001; the Alouette-R test Lefavrais, 2005) and reading fluency tests (Math Fluency subtest of the Woodcock-Johnson Test of achievement; WJ III Woodcock et al., 2001; the Alouette-R test Lefavrais, 2005), but with standardization norms adapted to their ages. In the Math Fluency subtest, participants solve simple addition, subtraction, and multiplication problems within a 3-min time limit. The test consists of 2 pages of 80 problems involving operands from 0 to 10. Addition, subtraction, and multiplication problems are intermixed, but multiplication problems are only introduced after Item 60. In the Alouette-R test, participants have to read a 265-word text aloud in 3 min. The number of words read and the number of pronunciation errors is used to calculate indices of reading speed and reading accuracy, respectively.

### 3.3 Measures at T2 (end of lockdown)

Towards the end of the first 2020 COVID-related lockdown in France, we contacted the families who participated in lab testing at T1. Parents were asked to fill out an online questionnaire from home. After asking for any change in SES situation since T1 as well as working conditions (e.g., loss of salary, possibilities of working from home) and material conditions (e.g., number of square meters per person living in the household, access to computer equipment) during lockdown, the questionnaire asked parents on both children's engagement in learning activities at home during lockdown and parental confidence towards academic domains (for a detailed list of items in English translated from original French and their descriptive statistics, see S2 Table).

Three survey items were used to measure children's engagement in learning activities. First, parents estimated children overall daily learning time with a 7-point rating scale (Less than 1 h per day; From 1 to 2 h per day; From 2 to 3 h per day; From 3 to 4 h per day; From 4 to 5 h per day; From 5 to 6 h per day, More than 6 h per day; *M* = 3.12 h). Second, parents estimated the time they spent themselves every day helping and/or supervising their children with homeschooling using a 10-point rating scale (Never; Less than 15 min per day; From 15 to 30 min per day; From 30 min to 1 h per day; From 1 to 2 h per day; From 2 to 3 h per day; From 3 to 4 h per day; From 4 to 5 h per day; From 5 to 6 h per day, More than 6 h per day; *M* = 1.87 h). Third, parents indicated how often they asked their children for extra homework related to arithmetic or to reading with a 7-point rating scale (Did not occur/Activity is not relevant to my child; Very rarely; 1–3 times per month; Once per week; 2–4 times per week; Almost daily; Daily).

We then measured parental confidence towards academic domains. This was done by asking parents to indicate how confident they felt about effectively supporting their child in homeschooling with a 3-point rating scale (Yes, I feel confident to help my child effectively; It is not easy, but I try to help my child as best I can; No, I do not feel confident to help my child effectively).

Finally, parents rated their overall and specific (professional and family related) levels of stress, using numerical scales from 0 to 10 (0 = no stress, 10 = maximum stress) (Lesage et al., 2009; Rolland et al., 2020). Children also completed the Revised Children's Manifest Anxiety Scale (RCMAS, Gerard and Reynolds, 2004). The RCMAS is a 37-item self-administered questionnaire that provides a quantitative assessment of children anxiety. Children were asked to describe on a 2-points rating scale (yes or no) their current feeling toward a large variety of statements (e.g., “I have difficulty falling asleep at night”, “I am always kind”, “I worry about what will happen”) We calculated a total anxiety score and three different sub-scores related to physiological anxiety, anxiety/hypersensitivity, and social concerns.

Finally, children's math and reading skills were assessed via online tasks, programmed using the Psychopy software (https://www.psychopy.org) and hosted on the Pavlovia platform (https://pavlovia.org). Math skills were assessed using an arithmetic task in which children were presented with a series of addition, subtraction, and multiplication problems. In each trial, a problem was presented for 800 ms at the center of screen. After an interval of 200 ms, a proposed answer was shown for 800 ms. Children had to indicate whether the answer was valid or invalid as quickly and as accurately as possible by pressing one of two keys on the computer keyboard. There were 60 trials in total (i.e., 12 addition problems, 24 subtraction problems, and 24 multiplication problems). The answer was valid in half of the trials and invalid in the other half. Trials included a variety of problems of various difficulties (see S3 Table for a full list of problems and answers). Trials were randomly presented. Reading skills were assessed using a rhyming task in which children were presented with pairs of written words. In each trial, the first word was presented for 800 ms at the center of screen. After an interval of 200ms, the second word was shown for 800 ms. Children had to indicate whether the words rhymed or not as quickly and as accurately as possible by pressing one of two keys on the computer keyboard. Words could have (1) similar orthography and similar phonology (e.g., “main” and “nain”), (2) similar orthography and different phonology (e.g., “fille” and “ville”), (3) different orthography and similar phonology (e.g., “train” and “pin”), and (4) different orthography and different phonology (e.g., “date” and “saut”). There were 48 trials in total, with 12 trials of each orthography and phonology combination (see S4 Table for a full list of word pairs). Trials were randomly presented. Two children did not complete these tasks (*n* = 50). The sample size for all analyses is therefore *n* = 52 subjects, except for those involving child scores at T2 (*n* = 50). All data, as well as the parental questionnaire at T1 and T2 can be found on OSF (https://osf.io/r8h65/).

### 3.4 Data analysis

We used bivariate Pearson correlations to test whether children's engagement in learning activities during lockdown (T2) as well as parental confidence toward homeschooling during lockdown (T2) increased with different family characteristics measured prior lockdown (T1). Partial correlations and regression models were used to control for a variety of either parental or children characteristics. All analyses were conducted in Jamovi version 1.6 (Jamovi, 2021).

## 4 Results

### 4.1 Children's arithmetic and reading scores

Children's accuracy on the online arithmetic task was positively associated with children's scores on the Math fluency subtest at T1 (*r* (48) = .470, *p* < .001). Children's accuracy on the online reading task at T2 was also positively associated with children's accuracy scores on the Alouette test at T1 (*r* (48) = .319, *p* = .024). Therefore, accuracy on the online arithmetic and reading tasks was converted into z-scores and used in the current analyses to control for children's math and reading skills in the main analyses below.

### 4.2 Physical and emotional environment of families during lockdown (T2)

At T2, most of the parents reported working from home (44%). A third of them stopped working completely (33%), a minority (8%) saw their working activity reduced (partial unemployment), and 15% continued to physically work at their regular workplace. The economic impact of the lockdown was relatively diverse: 30% of parents stated that the lockdown had no consequence for their budget, while 23% experienced a reduction in their budget. Only 4% of parents reported that the lockdown could potentially put them in a precarious situation.

Parent-average level of perceived personal and professional stress was relatively low but there were important individual differences (perceived personal stress, *M* = 4.08, *SD* = 2.61, range = 0–10; perceived professional stress, *M* = 3.60, *SD* = 2.81, range = 0–8). Children anxiety scores were average, but also had important interindividual differences (total anxiety score, *M* = 46.84, *SD* = 31.93, range = 1–99; social concerns, *M* = 49.14, *SD* = 26.73, range = 11–99; anxiety/hypersensitivity, *M* = 48.20, *SD* = 31.59, range = 4–99; physiological anxiety; *M* = 45.73, *SD* = 28.22, range = 4–93).

### 4.3 Relations between children's engagement in learning activities during lockdown (T2) and characteristics of the home learning environment (activities and expectations) prior lockdown (T1)

Children's engagement in learning activities during lockdown was measured using three survey items: (1) overall daily learning time, (2) daily time spent by parents helping children, (3) frequency of extra math work, and (4) frequency of extra reading homework. We anticipated that all of these outcome variables would be positively associated with characteristics of the home learning environment (activities and expectations) prior lockdown.

Overall, daily learning time (*M* = 3.12 h, *SD* = 1.25) was reduced compared to the length of a normal school day (5.5 h). It was also correlated with daily time spent by parents helping children (*r* (50) = .531, *p* < .001). Children who spent the most time working were also those who received extra reading (r (50) = .301, *p* = .015) and arithmetic homework (*r* (50) = .371, *p* = .003) from their parents. Bivariate correlations between these three output variables measured at T2 and family characteristics measured at T1 are shown in Table 1.

**Table 1:** Pearson correlation coefficients between children's engagement in learning activities during lockdown (T2) and family characteristics prior lockdown (T1).

	1.	2.	3.	4.	5.	6.	7.	8.	9.	10.
1. Overall daily learning time (T2)										
2. Daily time spent by parents helping children (T2)	0.53∗∗∗									
3. Frequency of extra math homework (T2)	0.37∗∗	0.25†								
4. Frequency of extra reading homework (T2)	0.30∗	0.29∗	0.65∗∗∗							
5. Home Numeracy Activities (T1)	0.12	0.28∗	0.24†	0.16						
6. Home Literacy activities (T1)	0.04	0.35∗	0.26†	0.41∗∗	0.59∗∗∗					
7. Parental expectations toward math (T1)	0.27∗	0.22	0.36∗∗	0.43∗∗	0.14	0.13				
8. Parental expectations toward literacy (T1)	0.28∗	0.27†	0.29∗	0.45∗∗∗	0.02	0.16	0.63∗∗∗			
9. Parental math attitude (T1)	0.01	-0.33∗	0.01	0.01	-0.09	-0.09	0.38∗∗	0.17		
10. Parental literacy attitude (T1)	0.11	0.05	0.09	0.27†	0.06	0.19	0.44∗∗∗	0.43∗∗	0.08	-

*N* = 52; ∗∗∗, *p* < .001; ∗∗, *p* < .01; ∗, *p* < .05, †, p < .1. P values are two-tailed.

Consistent with expectations, overall daily learning time during lockdown increased with parental expectations towards numeracy and literacy measured two years earlier. Regression analyses (see S5 Table) showed that parental expectations towards numeracy (SC = 0.368, 95% CI = [0.068, 0.667], t = 2.481, p = 0.017, η^2^p = 0.133) and literacy (SC = 0.471, 95% CI = [0.156, 0.786], t = 3.024, p = 0.004, η^2^p = 0.186) remained positively associated with overall daily learning time after adjusting for several potential confounding parental (education and income, level of personal stress, skills measured at T1), children (anxiety, access to digital devices, skills measured at T2) and home (physical space for each member of the household) factors*.*

Also consistent with expectations, there was a positive correlation between daily time spent by parents helping children and the frequency of home learning activities involving numeracy and literacy two years earlier. Regression analyses (see S6 Table) showed that the frequency of home learning activities at T1 remained positively associated with daily time spent by parents helping children at T2 after adjusting for confounding parental (education and income, level of personal stress, skills measured at T1), children (anxiety, access to digital devices, skills measured at T2) and home (physical space for each member of the household) factors (numeracy activities: SC = 0.328, 95% CI = [0.020, 0.635], t = 2.153, p = 0.037**,** η^2^p = 0.104 and literacy activities: SC = 0.342, 95% CI = [0.037, 0.647**],** t = 2.266, p = 0.029**,** η^2^p = 0.114).

Finally, children extra arithmetic and reading homework at T2 increased with parental expectations towards numeracy and literacy at T1. Extra homework at T2 also tended to increase with numeracy and literacy learning activities at T1. Regression analyses (see S7 Table) showed that children extra arithmetic and reading homework at T2 remained associated with parental expectations towards numeracy **(**SC = 0.333, 95% CI = [0.003, 0.663**],** t = 2.037, p = 0.048, η^2^p = 0.094**)** and literacy (SC = 0.538, 95% CI = [0.245, 0.832], t = 3.707, p < .001, η^2^p = 0.256) at T1 after adjusting for parental education and income, children's anxiety, children access to digital devices, physical space for each member of the household, parental level of perceived personal stress, children's skills measured at T2 and parents' skills measured at T1.

### 4.4 Relations between parental confidence about homeschooling during lockdown (T2) and characteristics of the home learning environment (parental expectations and attitudes) prior lockdown (T1)

Bivariate correlations between parental confidence in their ability to support their child during lockdown and family characteristics measured at T1 are shown in Table 2. Parental confidence increased with both parental attitude towards numeracy and literacy measured two years earlier. It also increased with parental expectations towards numeracy and literacy two years earlier. Regression analyses (see S8 and S9 Table) showed that these relations remained significant after adjusting for parental education and income, children's anxiety, children access to digital devices, physical space for each member of the household, parental level of perceived personal stress, parents' arithmetic and reading skills measured at T1, children's arithmetic (expectations toward numeracy: SC = 0.402, 95% CI = [0.117, 0.687], t = 2.847, p = 0.007, η^2^p = 0.168 and literacy SC = 0.421, 95% CI = [0.110, 0.732], t = 2.736, p = 0.009, η^2^p = 0.158; attitudes toward numeracy: SC = 0.321, 95% CI = [0.014, 0.627], t = 2.116, p = 0.041**,** η^2^p = 0.101). Note, however, that the positive relation between parental confidence and parental attitude towards literacy was no longer significant after adjusting for children reading skills measured at T2 (attitude toward literacy: SC = 0.367, 95% CI = [0.081, 0.653], t = 2.588, p = 0.013**,** η^2^p = 0.135).

**Table 2:** Pearson correlation coefficients between parental confidence about homeschooling during lockdown (T2) and family characteristics prior lockdown (T1).

	1.	2.	3.	4.	5.	6.	7.
1. Parental confidence in their ability to help their child for homeschool (T2)							
2. Home Numeracy Activities (T1)	0.01						
3. Home Literacy activities (T1)	-0.09	0.59∗∗∗					
4. Parental expectations toward math (T1)	0.37∗∗	0.14	0.13				
5. Parental expectations toward literacy (T1)	0.40∗∗	0.02	0.16	0.63∗∗∗			
6. Parental math attitude (T1)	0.42∗∗	-0.09	-0.09	0.38∗∗	0.17		
7. Parental literacy attitude (T1)	0.31∗	0.06	0.19	0.44∗∗	0.43∗∗	0.08	-

*N* = 52; ∗∗∗, *p* < .001; ∗∗, *p* < .01; ∗, *p* < .05. P values are two-tailed.

## 5 Discussion

Previous studies have shown a relation between parental adaptation to homeschooling during COVID-related lockdown and family SES (Bacher-Hicks et al., 2021; Bol, 2020; Jæger and Blaabæk, 2020; Reimer et al., 2021). SES, however, is a relatively distal factor that does not necessarily capture the specific family characteristics influencing adaptation to homeschooling. Here we aimed to study how parental response to homeschooling during lockdown was influenced by prior parental beliefs and attitudes towards academic learning as well as prior familiarity with literacy and numeracy activities at home. The present study relies on a longitudinal design in which families who participated in a previous study on the home learning environment two years before lockdown were contacted again at the end of the first 2020 French lockdown to measure parental adaptation to homeschooling at that time. This allowed us a explore the relations between a range of measures and this adaptation.

### 5.1 How prior parental attitudes and expectations as well as prior shared activities influence parental adaptation to homeschooling during lockdown

Consistent with previous studies conducted in other countries (e.g. United-States, Domina et al., 2021; Gao et al., 2020; Czech Republic, Brom et al., 2020; England, Andrew et al., 2020; Germany Grewenig et al., 2020 and Spain, Bonal & González, 2020), we found that French children cut their daily work time in half (compared to a normal school day) during the lockdown. However, the time spent doing homework was variable between families. Notably, it increased with prior parental expectations towards academic learning (both in terms of numeracy and literacy). The time spent doing homework with parents also increased with the prior frequency of both home numeracy and literacy activities measured two years earlier. Finally, we found that parental confidence about homeschooling during lockdown increased with parental expectations and attitudes towards academic learning measured two years earlier.

Overall, these findings are consistent with the idea that parental attitudes and academic expectations as well as shared literacy and numeracy practices between parents and children define what is commonly called the “home learning environment” (Elliott et al., 2019; Elliott and Bachman, 2018). These aspects are likely to be related, as a number of previous studies have found a positive relation between parental expectations regarding children's academic achievement and the frequency of home learning practices (Galindo and Sheldon, 2012, Kleemans et al., 2012, LeFevre et al., 2002, LeFevre et al., 2010, Martini and Sénéchal, 2012, Musun-Miller and Blevins-Knabe, 1998, Silver et al., 2021, Skwarchuk et al., 2014, Susperreguy et al., 2020). Our results suggest that characteristics such as the frequency of shared practices at home, but also parental attitudes and expectations towards academic learning, might be relatively stable measures that may continue to predict the home learning environment several years later, even in unprecedented conditions that disrupt the left of both parents and children.

### 5.2 Relations between prior home learning environment and family adaptation to homeschooling during lockdown are not accounted for by a range of background variables

Critically, our study is relatively unique in that we measured a range of background characteristics in both children and parents. This makes it possible to dissociate the effect of home learning characteristics from other effects that may also affect children learning time. We discuss here three of these potential confounds. First, several previous studies of homeschooling during COVID-related lockdowns have found an effect of family SES on time spent working by the child (Bonal and González, 2020; Domina et al., 2021; Gao et al., 2020). Here, we found that the relation between parental expectations and daily learning time was independent from SES, as it remained significant even after controlling for parental income and education. Second, the relations between several aspects of the home learning environment and daily learning time were also not dependent upon material characteristics of the home environment, such as children's access to digital devices and physical space for each member of the household. This suggests that homeschooling conditions may not necessarily depend on financial or material conditions, but rather on the quality and quantity of interactions between parents and children around numeracy and literacy. Third, our analyses also controlled for differences in parent's and children's anxiety scores. This is noteworthy because mental health of both parents and children may be affected by lockdown (Kubb and Foran, 2020). For example, it has been suggested that being a parent increases the risk of experiencing significant stress during lockdown (Achterberg et al., 2020; Brown et al., 2020; Patrick et al., 2020). Levels of child's anxiety during the lockdown have also been shown to correlate with the size of the learning loss (Di Pietro et al., 2020). Here, we show that the relations between the home learning environment and parental adaptation to homeschooling during lockdown were largely independent from anxiety levels.

### 5.3 Limitations

To our knowledge, our study is the first to assess the relations between the home learning environment and homeschooling conditions during lockdown, while controlling for a wide range of factors. However, it is important to acknowledge at least three limitations. A first limitation is that our measure of the homeschooling was an electronic questionnaire sent by email to parents and children. A drawback of this approach is that families without sufficient internet connection or computer equipment would not have been able to participate. The use of a questionnaire also increases the risk of social desirability bias among parents (King and Bruner, 2000). Second, although our sample includes families from diverse socio-economic backgrounds, it is constrained by the number of families who participated in our previous experiment and is therefore limited in size. Not only does this make it possible that we might have lacked the power to detect true effects, it is also possible that the effect sizes found might have been overestimated (Button et al., 2013). Nonetheless, we think that the present data are valuable and might inform future meta-analyses examining the predictors of adaptation to school closures across different countries and larger samples. Third, our results are only descriptive and correlational. In other words, although our results are consistent with the idea that home learning environment factors may inform on how families adapt to homeschooling situation due to lockdown, it is also possible that other non-measured factors drive this relation. We attempted to minimize this limitation by measuring and accounting for various potential confounding factors. However, it is clear that there might be a number of factors that were not accounted for.

## 6 Conclusion

In sum, our study demonstrates that investigating several aspects of the home learning environment may provide valuable information regarding homeschooling conditions during an unexpected social crisis such that the COVID pandemic. Critically, this information goes above and beyond a mere examination of differences in SES between families. Although our results are correlational in nature, they also suggest that parental attitudes and expectations may be promising targets for future interventions aiming to improve the home learning environment of children, particularly in the context of the current crisis.

## Declarations

### Author contribution statement

Cléa Girard: Conceived and designed the experiments; Performed the experiments; Analyzed and interpreted the data; Wrote the paper.

Jérôme Prado: Conceived and designed the experiments; Wrote the paper.

### Funding statement

This research did not receive any specific grant from funding agencies in the public, commercial, or not-for-profit sectors.

### Data availability statement

Data associated with this study has been deposited at https://osf.io/r8h65/?view_only=738e8684565945eb9174ab67275d0607.

### Declaration of interests statement

The authors declare no conflict of interest. This research was supported by grants from the Agence Nationale de la Recherche (ANR-14-CE30-0002 and ANR-17-CE28-0014) to JP.

### Additional information

No additional information is available for this paper.

## Appendix A Supplementary data

Supplemental Materials_revised Supplemental Materials_revised

Parental-Questionnaire-Covid-Lockdown-T2-English.pdf Parental-Questionnaire-Covid-Lockdown-T2-English.pdf

Parental-questionnaire-Covid-Lockdown-T2.pdf Parental-questionnaire-Covid-Lockdown-T2.pdf

Parental-questionnaire-T1.pdf Parental-questionnaire-T1.pdf

Parental-questionnaire-T1-English-translation.pdf Parental-questionnaire-T1-English-translation.pdf
