# Supplementary material for: Prior home learning environment is associated with adaptation to homeschooling during COVID lockdown
Source: Heliyon. 2022 Apr 19;8(4):e09294. doi: 10.1016/j.heliyon.2022.e09294 (PMC9017091; doi:10.1016/j.heliyon.2022.e09294)
Supplement: Supplemental Materials_revised [file mmc1.docx]

| **S1 Table.** Detailed items for questionnaire at T1. | | | | |
| --- | --- | --- | --- | --- |
|  | **Item** | **Mean (SD)**  **or n** | **Min** | **Max** |
| **Socio-Economic information** |  |  |  |  |
|  | **Parental education** | 3.05 (2.36) | -7 | 8 |
|  | primary | n=1 |  | - |
|  | secondary | n=8 |  | - |
|  | undergraduate | n=33 |  | - |
|  | master or higher | n=24 |  | - |
|  | **Monthly income^4^** | 1 727  (1 078) | 500 | 5 500 |
|  | €0-1,000 | n=18 |  |  |
|  | €1,000-2,000 | n=25 |  |  |
|  | €2,000-3,000 | n=16 |  |  |
|  | €3,000-4,000 | n=5 |  |  |
|  | €4,000-5,000 | n=1 |  |  |
|  | €5,000-6,000 | n=1 |  |  |
|  |  |  |  |  |
| **Home Numeracy activities**  (36 items) |  | 1.48 | 0 | 5 |
|  | **Informal** |  |  |  |
|  | Weighting / counting when shopping^2^ | 0.94 (1.03) | 0 | 5 |
|  | Counting out money^1^ | 1.2 (1.05) | 0 | 4 |
|  | Paying for shopping^2^ | 1.06 (1.09) | 0 | 5 |
|  | Comparing magnitudes^1^ | 1 (1.03) | 0 | 4 |
|  | Playing board games with a numerical dice^1^ | 1.29 (0.83) | 0 | 5 |
|  | Playing number card games^1^ | 0.92 (0.82) | 0 | 5 |
|  | Playing computer / tablet games involving numbers^1^ | 0.71 (0.75) | 0 | 3 |
|  | Making/sorting collections^1^ | 0.68 (0.86) | 0 | 4 |
|  | Measuring lengths/widths^1^ | 0.8 (0.7) | 0 | 3 |
|  | Talking about temperature or speed^2^ | 3 (1.54) | 0 | 5 |
|  | Measuring speeds^1^ | 2.65 (1.6) | 0 | 5 |
|  | Using a calculator^1^ | 0.76 (1.02) | 0 | 5 |
|  | Measuring ingredients while cooking^1^ | 1.44 (0.99) | 0 | 5 |
|  | Talking about the time with a watch or a clock^1^ | 3.77 (1.4) | 1 | 5 |
|  | Talking about the date with a calendar^1^ | 2.98 (1.53) | 0 | 5 |
|  | Dialing phone numbers^2^ | 1.06 (1.37) | 0 | 5 |
|  | Singing songs with numbers^2^ | 0.79 (0.86) | 0 | 5 |
|  | **Basic** |  |  |  |
|  | Counting objects^2^ | 1.74 (1.44) | 0 | 5 |
|  | Counting without objects^2^ | 1.03 (0.94) | 0 | 4 |
|  | Memorizing results of simple addition problems^2^ | 1.70 (1.38) | 0 | 5 |
|  | Memorizing multiplication tables^2^ | 2.03 (1.4) | 0 | 5 |
|  | Comparing quantities^2^ | 1.86 (1.54) | 0 | 5 |
|  | Adding numbers^2^ | 2.38 (1.3) | 0 | 5 |
|  | Subtracting single-digit numbers (e.g., 8-1) ^2^ | 2.41 (1.51) | 0 | 5 |
|  | Multiplying single-digit numbers (e.g., 2x3) ^2^ | 2.26 (1.34) | 0 | 5 |
|  | Talking about sharing^1^ | 2.39 (1.81) | 0 | 5 |
|  | Dividing small numbers (e.g., 6÷2) ^2^ | 1.17 (1.29) | 0 | 5 |
|  | Writing numbers up to 20^2^ | 1.14 (0.92) | 0 | 5 |
|  | Writing numbers up to 100^2^ | 1.17 (0.98) | 0 | 5 |
|  | Reading numbers up to 20^2^ | 1 (1.13) | 0 | 5 |
|  | Reading numbers up to 100^2^ | 1.21 (1.05) | 0 | 5 |
|  | **Advanced** |  |  |  |
|  | Subtracting double-digit numbers (e.g., 34-16) ^2^ | 1.52 (1.42) | 0 | 5 |
|  | Multiplying double-digit numbers (e.g., 12x6) ^2^ | 0.94 (1.09) | 0 | 4 |
|  | Dividing double-digit numbers (e.g., 12÷4) ^2^ | 0.29 (0.62) | 0 | 3 |
|  | Writing numbers up to 1,000^2^ | 0.89 (0.94) | 0 | 4 |
|  | Reading numbers up to 1,000^2^ | 1.12 (1.01) | 0 | 5 |
|  |  |  |  |  |
| **Home Literacy practices**  (18 items) |  | 2.07 | 0 | 5 |
|  | **Informal** |  |  |  |
|  | Telling invented stories^2^ | 1.15 (1.22) | 0 | 5 |
|  | Talking about school day^2^ | 4.64 (0.57) | 3 | 5 |
|  | Playing computer/tablet games involving reading or spelling^1^ | 0.65 (0.83) | 0 | 4 |
|  | Reading texts in everyday life (advertisement, etc.) ^2^ | 3.71 (1.31) | 0 | 5 |
|  | Visiting the library for children’s books^1^ | 1.24 (0.8) | 0 | 5 |
|  | Singing songs^2^ | 2.91 (1.61) | 0 | 5 |
|  | Singing alphabet songs^2^ | 0.89 (0.78) | 0 | 5 |
|  | Making up rhymes^2^ | 1.39 (1.2) | 0 | 5 |
|  | **Basic** |  |  |  |
|  | Writing short texts^2^ | 0.85 (0.8) | 0 | 4 |
|  | Reading short texts^2^ | 3.03 (1.62) | 1 | 5 |
|  | Listening while my child reads books out loud^1^ | 2.55 (1.51) | 0 | 5 |
|  | Reading together^1^ | 2.14 (1.37) | 0 | 5 |
|  | **Advanced** |  |  |  |
|  | Writing long texts^2^ | 0.36 (0.54) | 0 | 2 |
|  | Reading long texts^2^ | 2.2 (1.46) | 0 | 5 |
|  | Asking questions when my child reads alone ^2^ | 2.35 (1.43) | 0 | 5 |
|  | Asking questions when we read together^1^ | 1.8 (1.35) | 0 | 5 |
|  | Teaching and correcting spelling^2^ | 2.98 (1.38) | 0 | 5 |
|  | Teaching and correcting conjugation^2^ | 2.33 (1.28) | 0 | 5 |
|  |  |  |  |  |
| **Parental expectations toward numeracy** (13 items) |  | 2.07 | -1 | 3 |
|  | **Basic** |  |  |  |
|  | Count up to 100^1^ | 2.27 (.93) | -1 | 3 |
|  | Count up to 1,000^1^ | 1.94 (1.04) | -1 | 3 |
|  | Read numbers up to 100^1^ | 2.20 (0.89) | -1 | 3 |
|  | Read numbers up to 1,000^1^ | 2.02 (1.02) | -1 | 3 |
|  | Know simple sums without counting on fingers (e.g., 2+2) ^1^ | 2.36 (0.75) | -1 | 3 |
|  | Know how to solve complex addition problems (e.g., 15+12) ^1^ | 2 (0.78) | 1 | 3 |
|  | Know simple multiplication problems (e.g., 2x6) ^1^ | 2.32 (0.70) | 1 | 3 |
|  | **Advanced** |  |  |  |
|  | Know complex multiplication problems  (e.g., 14x7) ^2^ | 1.85 (.96) | -1 | 3 |
|  | Know fractions and how to use them (e.g., 2/3) ^2^ | 1.23 (1.16) | -1 | 3 |
|  | Know how to solve division problems  (e.g., 30÷5) ^2^ | 1.72 (0.98) | -1 | 3 |
|  | Know decimal numbers (e.g., 3.2) ^2^ | 1.38 (1.00) | -1 | 3 |
|  | Know how to calculate with decimal numbers^2^ | 1.12 (1.07) | -1 | 3 |
|  | Know probabilities and how to use them^2^ | 0.02 (1.33) | -3 | 2 |
| **Parental expectations toward literacy** (12 items) |  | 2.01 (0.71) | -3 | 3 |
|  | **Basic** |  |  |  |
|  | Write in a fluent and efficient manner^2^ | 2.23 (0.96) | -1 | 3 |
|  | Write a text of half a page^2^ | 1.70 (1.03) | -1 | 3 |
|  | Spell correctly common words^2^ | 2.47 (0.79) | -1 | 3 |
|  | Read simple picture books^1^ | 2.42 (0.77) | -1 | 3 |
|  | Read and understand short paragraphs^2^ | 2.54 (0.59) | 1 | 3 |
|  | **Advanced** |  |  |  |
|  | Use writing autonomously (e.g. keep track of reading) ^2^ | 1.59 (1.09) | -3 | 3 |
|  | Spell correctly invariable words^2^ | 2.40 (0.63) | 1 | 3 |
|  | Read chapter books (i.e. books with words only)^1^ | 2.02 (0.96) | -1 | 3 |
|  | Read an entire book^2^ | 2.06 (1.01) | -1 | 3 |
|  | Understand and interpret texts^2^ | 1.71 (0.92) | -1 | 3 |
|  | Make an oral presentation^2^ | 1.61 (0.96) | -1 | 3 |
|  | Ability to engage in structured spoken interaction (e.g., debate) ^2^ | 1.74 (1.03) | -1 | 3 |
| **Parental attitudes toward math** (6 items) |  | 0.64 (0.58) | -1.5 | 1.5 |
|  | “When I was at school, I was good at math” ^1^ | 0.54 (1.11) | -1.5 | 1.5 |
|  | “My job involves using math” ^1^ | 0.15 (1.13) | -1.5 | 1.5 |
|  | “I find math enjoyable” ^1^ | 0.46 (1.06) | -1.5 | 1.5 |
|  | “I don’t avoid situations that involve math” ^1^ | 0.94 (0.86) | -1.5 | 1.5 |
|  | “I am confident about engaging my child in a math activity” ^2^ | 0.77 (0.75) | -1 | 1.5 |
|  | “In my home, math skills are taught randomly throughout the day.” ^2^ | 0.95 (0.65) | -1.5 | 1.5 |
| **Parental attitudes toward literacy** (6 items) |  | 0.98 (0.40) | -1.5 | 1.5 |
|  | “When I was at school, I was good at literacy” ^1^ | 1.12 (0.67) | -1.5 | 1.5 |
|  | “My job involves using literacy” ^1^ | 0.50 (1.09) | -1.5 | 1.5 |
|  | “I find literacy enjoyable” ^1^ | 1.13 (0.50) | -1 | 1.5 |
|  | “I don’t avoid situations that involve literacy” ^1^ | 1.12 (0.63) | -1.5 | 1.5 |
|  | “I am confident about engaging my child in a literacy activity” ^2^ | 1.06 (0.55) | -1 | 1.5 |
|  | “In my home, literacy skills are taught randomly throughout the day.” ^2^ | 0.95 (0.70) | -1.5 | 1.5 |
|  |  |  |  |  |

***Notes. N*=66**

**Activities Rating**: Did not occur/Activity is not relevant to my child was coded 0, Child is doing the activity without parent was coded 1, Parents used to engage in the activity in the past was coded 1, 1-3 times per month was coded 1, Once per week was coded 2, 2-4 times per week was coded 3, Almost daily was coded 4, Daily was coded 5.

**Expectations Rating:** Really not important was coded -3, Not important was coded -1, No opinion was coded 0, Important was coded 1, Very important was coded 2, Extremely important was coded 3.

**Attitudes Rating:** Strongly disagree was coded -1.5; Disagree was coded -1; Not sure was coded 0; Agree was coded 1; Strongly agree was coded 1.5

^1^Items directly translated from LeFevre et al.’s questionnaire.

^2^Items adapted from LeFevre et al.’s questionnaire to account for the fact that children in the present study are older.

| **S2 Table.** Detailed items for questionnaire at T2. | | | | |
| --- | --- | --- | --- | --- |
|  | **Item** | **Mean (SD)**  **or n** | **Min** | **Max** |
| **Learning time during covid lockdown** |  |  |  |  |
|  | **Child overall daily learning time** | 3.12 hours (1.25) | 1.5 | 5.5 |
|  | Less than 1 hour | n=0 |  |  |
|  | Between 1 and 2 hours | n=9 |  |  |
|  | Between 2 and 3 hours | n=19 |  |  |
|  | Between 3 and 4 hours | n=14 |  |  |
|  | Between 4 and 5 hours | n=4 |  |  |
|  | Between 5 and 6 hours | n=5 |  |  |
|  | More than 6 hours | n=1 |  |  |
|  | **Parental daily time spent helping their child** | 1 87 hours  (1.40) | 0.13 | 5.5 |
|  | Never | n=0 |  |  |
|  | Less than 15 minutes per day | n=4 |  |  |
|  | Between 15 and 30 minutes per day | n=5 |  |  |
|  | Between 30 minutes and 1 hour per day | n=12 |  |  |
|  | Between 1 and 2 hours | n=9 |  |  |
|  | Between 2 and 3 hours | n=11 |  |  |
|  | Between 3 and 4 hours | n=6 |  |  |
|  | Between 4 and 5 hours | n=4 |  |  |
|  | Between 5 and 6 hours | n=1 |  |  |
|  | **Frequency of extra math homework** |  |  |  |
|  | Never | n=34 |  |  |
|  | Very rarely | n=0 |  |  |
|  | 1 to 3 times a month | n=3 |  |  |
|  | 1 time a week | n=10 |  |  |
|  | 2 to 4 times a week | n=3 |  |  |
|  | Almost every day | n=0 |  |  |
|  | Every day | n=2 |  |  |
|  | **Frequency of extra reading homework** |  |  |  |
|  | Never | n=34 |  |  |
|  | Very rarely | n=0 |  |  |
|  | 1 to 3 times a month | n=6 |  |  |
|  | 1 time a week | n=2 |  |  |
|  | 2 to 4 times a week | n=3 |  |  |
|  | Almost every day | n=1 |  |  |
|  | Every day | n=6 |  |  |
| **Parental emotion** |  |  |  |  |
|  | Parental confidence about effectively supporting their child in homeschooling | 1.85 (0.36) | 1 | 2 |
|  | Level of personal stress | 3.60 (2.81) | 0 | 8 |
|  | Level of professional stress | 4.08 (2.61) | 0 | 10 |
| **Home material conditions** |  |  |  |  |
|  | Square meters per person | 20.98 (6.07) | 11.67 | 45 |
|  | Child computer access | 1.52 (0.70) | 1 | 3 |
|  | Reduction of family income due to lockdown | 1.42 (0.91) | 0 | 3 |
|  | Risk of poverty | 0.54 (0.64) | 0 | 2 |

***Notes. N*=52**

**Parental confidence about effectively supporting their child in homeschooling** was coded 0 to 2.

**Level of personal and professional stress** was coded 0 to 10.

**Child computer access**: personal computer (or tablet) was coded 3, computer left at his disposal was coded 2, shared computer (with a parent or another child) was coded 1

**Reduction of family income due to lockdown** and **risk of poverty**: certainly not was coded 0, probably not was coded 1, probably was coded 2, very likely was coded 3

**Risk of poverty:** certainly not was coded 0, probably not was coded 1, probably was coded 2, very likely was coded 3

**S3 Table. List of stimuli for the online arithmetic decision task**

| **Operation** | **Complexity levels** | **Pairs of operands** | **Answers** | |  |
| --- | --- | --- | --- | --- | --- |
|  |  |  | **Valid** | **Invalid** |  |
|  |  | 2x3 | 6 | 4 |  |
|  |  | 2x4 | 8 | 10 |  |
|  |  | 2x5 | 10 | 15 |  |
|  | Easy | 3x4 | 12 | 16 |  |
|  |  | 3x5 | 15 | 20 |  |
|  |  | 4x5 | 20 | 25 |  |
|  |  | 6x7 | 42 | 36 |  |
| **Multiplication** |  | 6x8 | 48 | 56 |  |
|  | Advanced | 6x9 | 54 | 63 |  |
|  |  | 7x8 | 56 | 48 |  |
|  |  | 7x9 | 63 | 54 |  |
|  |  | 8x9 | 72 | 63 |  |
|  |  | 15-12 | 3 | 2 |  |
|  |  | 14-12 | 2 | 3 |  |
|  |  | 16-13 | 3 | 4 |  |
|  | Easy | 3-2 | 1 | 2 |  |
|  |  | 5-3 | 2 | 3 |  |
|  |  | 4-3 | 1 | 2 |  |
| **Subtraction** |  | 7-3 | 4 | 3 |  |
|  |  | 8-2 | 6 | 4 |  |
|  | Advanced | 9-4 | 5 | 6 |  |
|  |  | 18-15 | 3 | 2 |  |
|  |  | 16-11 | 5 | 6 |  |
|  |  | 19-13 | 6 | 5 |  |
|  |  | 5+8 | 13 | 12 |  |
|  |  | 4+13 | 17 | 15 |  |
|  | Zareki’ addition | 9+7 | 16 | 15 |  |
| **Addition** | items | 15+12 | 27 | 29 |  |
|  |  | 13+19 | 32 | 34 |  |
|  |  | 17+25 | 42 | 43 |  |

**S4 Table. List of stimuli for the online rhyme task (reading)**

| **Phonology** | **Orthography** | **word pairs** | **Phonology** | **Orthography** | **word pairs** |
| --- | --- | --- | --- | --- | --- |
| **Similar** | **Similar** | presse - tresse | **Different** | **Similar** | tabac - hamac |
|  |  | main - nain |  |  | donc - tronc |
|  |  | tard - lard |  |  | virus - intrus |
|  |  | rouille - fouille |  |  | choc - croc |
|  |  | miel - ciel |  |  | fille - ville |
|  |  | local - bocal |  |  | amer - ramer |
|  |  | table - sable |  |  | mille - quille |
|  |  | vent - dent |  |  | sang - gang |
|  |  | sueur - lueur |  |  | hiver - boucher |
|  |  | folle - colle |  |  | dix - prix |
|  |  | peau - seau |  |  | fusil - avril |
|  |  | sac -lac |  |  | cours- ours |
|  | **Different** | chat - villa |  | **Different** | gain - rail |
|  |  | terre - fer |  |  | brosse- botte |
|  |  | pain - faim |  |  | ile - ruelle |
|  |  | sous - doux |  |  | part - tir |
|  |  | soleil -oreille |  |  | dire - froid |
|  |  | cou -loup |  |  | bas - pate |
|  |  | corps - bord |  |  | role - roux |
|  |  | dos - taux |  |  | cas - sport |
|  |  | place - basse |  |  | date - saut |
|  |  | train - pin |  |  | fort - vitre |
|  |  | bois - poids |  |  | beau - madame |
|  |  | salle - ovale |  |  | ile- ruelle |

| **S5 Table.** Effect sizes and t-values associated with multiple regression analyses of overall daily learning time on parental expectations towards numeracy and literacy | | | | |
| --- | --- | --- | --- | --- |
|  | Overall daily learning time on numeracy expectations | | Overall daily learning time on literacy expectations | |
|  | η²p | t | η²p | t |
| Parental expectations for numeracy | **0.133** | **2.481** | - | - |
| Parental expectations for literacy | - | - | **0.186** | **3.024** |
| Parental education | 0.069 | -0.723 | 0.075 | -1.798 |
| Parental income | 0.008 | 0.563 | 0.023 | 0.980 |
| Parental personal stress | 0.028 | 1.070 | 0.039 | 1.279 |
| Parental score at T1 in the domain | **0.109** | **2.207** | **0.102** | **2.128** |
| Child anxiety | 0.034 | -1.188 | 0.051 | -1.471 |
| Child access to digital devices | **0.160** | **2.762** | **0.155** | **2.710** |
| Child score at T2 in the domain | 0.008 | -0.562 | 0.009 | -0.598 |
| Physical space | 0.017 | -0.844 | 0.030 | -1.115 |
|  |  |  |  |  |
| R^2^ | 0.329 | | 0.318 | |
| *N*=52 (*n*=50 for children score at T2); *p* < .05 (two-tailed) in bold; *η2p*s represent effect sizes that can be considered small (0.01), medium (0.06) or large (0.14). | | | | |

| **Table S6.** Effect sizes and t-values associated with multiple regression analyses of daily time spent by parents helping children | | | | |
| --- | --- | --- | --- | --- |
|  | Daily time spent helping children | | Daily time spent helping children | |
|  | η²p | t | η²p | t |
| Home numeracy activities | **0.104** | **2.153** | - | - |
| Home literacy activities | - | - | **0.114** | **2.266** |
| Parental education | 0.001 | -0.188 | 0.000 | -0.059 |
| Parental income | 0.005 | -0.432 | 0.000 | -0.012 |
| Parental personal stress | 0.088 | 1.959 | 0.060 | 1.603 |
| Parental score at T1 in the domain | **0.126** | **2.405** | 0.011 | 0.663 |
| Child anxiety | 0.007 | -0.549 | 0.009 | -0.600 |
| Child access to digital devices | 0.047 | 1.406 | 0.020 | 0.914 |
| Child score at T2 in the domain | 0.015 | -0.776 | 0.03 | -1.169 |
| Physical space | 0.016 | 0.805 | 0.000 | 0.101 |
|  |  |  |  |  |
| R^2^ | 0.292 | | 0.241 | |
| *N*=52 (*n*=50 for children score at T2); *p* < .05 (two-tailed) in bold; *η2p*s represent effect sizes that can be considered small (0.01), medium (0.06) or large (0.14). | | | | |

| **Table S7** Effect sizes and t-values associated with multiple regression analyses of extra arithmetic and reading homework at T2 on parental expectations towards numeracy and literacy at T1 | | | | |
| --- | --- | --- | --- | --- |
|  | Extra arithmetic homework on numeracy expectations | | Extra literacy homework on literacy expectations | |
|  | η²p | t | η²p | t |
| Parental expectations for numeracy | **0.094** | **2.037** | **-** | **-** |
| Parental expectations for literacy | - | - | **0.256** | **3.707** |
| Parental education | 0.006 | -0.504 | 0.042 | -1.319 |
| Parental income | 0.007 | -0.533 | 0.002 | -0.310 |
| Parental personal stress | 0.000 | -0.085 | 0.039 | 1.273 |
| Parental score at T1 in the domain | 0.001 | -0.176 | **0.111** | **0.232** |
| Child anxiety | 0.005 | -0.433 | **0.122** | **-2.360** |
| Child access to digital devices | 0.027 | -1.044 | 0.031 | 1.133 |
| Child score at T2 in the domain | 0.001 | 0.143 | 0.010 | 0.640 |
| Physical space | 0.001 | 0.196 | 0.003 | -0.340 |
|  |  |  |  |  |
| R^2^ | 0.184 | | 0.407 | |
| *N*=52 (*n*=50 for children score at T2); *p* < .05 (two-tailed) in bold; *η2p*s represent effect sizes that can be considered small (0.01), medium (0.06) or large (0.14). | | | | |

| **Table S8** Effect sizes and t-values associated with multiple regression analyses of parental confidence about homeschooling at T2 on parental expectations toward literacy and numeracy at T1 | | | | |
| --- | --- | --- | --- | --- |
|  | Parental confidence | | Parental confidence | |
|  | η²p | t | η²p | t |
| Parental expectations for numeracy | **0.168** | **2.847** | **-** | **-** |
| Parental expectations for literacy | - | - | **0.158** | **2.736** |
| Parental education | 0.002 | 0.316 | 0.000 | 0.115 |
| Parental income | 0.079 | -1.854 | 0.062 | -1.623 |
| Parental personal stress | **0.103** | **-2.138** | 0.062 | -1.625 |
| Parental score at T1 in the domain | **0.133** | **2.475** | 0.000 | -0.002 |
| Child anxiety | 0.009 | 0.606 | 0.003 | 0.343 |
| Child access to digital devices | 0.041 | 1.303 | 0.003 | 0.340 |
| Child score at T2 in the domain | 0.006 | 0.497 | 0.084 | 1.914 |
| Physical space | 0.012 | -0.710 | 0.016 | -0.817 |
|  |  |  |  |  |
| R^2^ | 0.392 | | 0.335 | |
| *N*=52 (*n*=50 for children score at T2); *p* < .05 (two-tailed) in bold; *η2p*s represent effect sizes that can be considered small (0.01), medium (0.06) or large (0.14). | | | | |

| **Table S9** Effect sizes and t-values associated with multiple regression analyses of parental confidence about homeschooling at T2 on parental attitudes toward literacy and numeracy at T1 | | | | |
| --- | --- | --- | --- | --- |
|  | Parental confidence | | Parental confidence | |
|  | η²p | t | η²p | t |
| Parental numeracy attitude | **0.101** | **2.116** | **-** | **-** |
| Parental literacy attitude | - | - | **0.135** | **2.588** |
| Parental education | 0.001 | 0.228 | 0.024 | 1.033 |
| Parental income | **0.093** | **-2.028** | 0.045 | -1.427 |
| Parental personal stress | 0.053 | -1.502 | 0.055 | -1.580 |
| Parental score at T1 in the domain | 0.083 | 1.902 | 0.015 | -0.806 |
| Child anxiety | 0.013 | 0.733 | 0.017 | -0.852 |
| Child access to digital devices | 0.007 | 0.522 | 0.014 | 0.790 |
| Child score at T2 in the domain | 0.045 | 1.375 | - | - |
| Physical space | 0.000 | 0.036 | 0.003 | -0.354 |
|  |  |  |  |  |
| R^2^ | 0.342 | | 0.273 | |
| *N*=52 (*n*=50 for children score at T2); *p* < .05 (two-tailed) in bold; *η2p*s represent effect sizes that can be considered small (0.01), medium (0.06) or large (0.14). | | | | |
