# Supplementary material for: Prior home learning environment is associated with adaptation to homeschooling during COVID lockdown
Source: Heliyon. 2022 Apr 19;8(4):e09294. doi: 10.1016/j.heliyon.2022.e09294 (PMC9017091; doi:10.1016/j.heliyon.2022.e09294)
Supplement: Parental-Questionnaire-Covid-Lockdown-T2-English.pdf [file mmc2.pdf]

Parental Questionnaire T2
Covid Lockdown
(English translation)

• Are you the mother or the father of the child in the study?

• Has your family and work situation changed since your previous participation? (outside of this period of COVID-19 lockdown): *Yes my family situation has changed ; Yes, my work situation has changed; Yes my family AND professional situation has changed ; No*

If yes :
What is the highest level of education you have now completed?
XXX

Which of these categories best describes your current work situation? *I am a student or in training; I have a full-time job; I have a part-time job; I am currently unemployed*

Which of these categories describes most accurately your current occupation? *Farmer; Craftsman, merchant and company manager; Managers and professionals in higher education Intermediate professions; Employee; Worker; Inactive (never worked); Other*

What is your current net monthly income? *0 € - 999 €;1,000 € - 1,999 €; 2,000 € - 2,999 €; 3,000 € - 3,999 €; 4,000 € - 4,999 €; 5,000 € - 5,999 €;6,000 € - 6,999 €;7,000 € - 7,999 €; 8,000 € - 8,999 €; 9,000 € - 9,999 €; 10,000 € et plus*

My family situation has changed: *Birth of a child; Marriage / new cohabitation; Separation; Other*

• During the lockdown period, are you in a home where you have access to: *at least one balcony/terrace; a private garden; sports facilities; none of the above*

• How many people are in your home (including you) during lockdown period: *1; 2; 3; 4; 5; 6; 7 or more*

• How many meters square do you approximately have in your home? *Less than 20 m^2 Between 20 and 40 m2 Between 40 and 60 m2 Between 60 and 80 m2 Between 80 and 100 m2 More than 100 m2*

• Does your child included in the study have a single room? *Yes; No*

• You live in*: An urban area (city center, town); A peri-urban area (suburbs of a metropolis, surroundings of a city); A rural area (countryside)*

• During this lockdown period, are you working? *yes I am teleworking; yes at my work site only; yes on my work site and at remote; yes but my activity is partly reduced (partial unemployment) no I am partially unemployed; no I am on sick leave; no I am on leave or on leave for childcare; no I am looking for a job.*

• Will this period of confinement affect your budget? *Very likely; probably; probably not; definitely not*

• Will you be at risk for a precarious situation? *Very likely; probably; probably not; definitely not*

• Outside of school vacations, during this period of confinement, how much time per day do you spend on each of these activities (on average over the week) ?

	Never	Less than 30 minutes	30 minutes to 1 hour	From 1 to 2 hours	From 2 to 3 hours	From 3 to 4 hours	From 4 to 5 hours	From 5 to 6 hours	More than 6 hours
Work and/or study									
Taking care of yourself (washing, dressing, hair, make-up...)									
Playing sports									
Taking care of your child (meal, bath, dressing,...)									
Playing with your child (board game, video game, ...)									
Working with your child (home school, extra homework, etc.)									
Doing creative activities with your child (music, drawing...)									
Cooking, DIY, housework/cleaning									
Watching the news									
Reading, watching TV (excluding news)									
Playing video games									
Ruminating on anxious thoughts									
Interacting on social networks, making phone calls, sending text messages									

• During the lockdown, did you feel able to efficiency guide your child through the school program? *Yes; Yes, but I had difficulties; No*

• During this lockdown, do you consider the amount of schoolwork required of your child to be : *Really too important; Too important; Quite adequate; Too small; Much too low*

• During this lockdown, how did you organize the school vacation period? *By trying to make it as similar as possible to the "usual" vacations: no school at all; By relaxing the rhythm of the previous weeks but continuing some school activities at home; By maintaining the school rhythm of the previous weeks*

• During this lockdown, have you ever asked your child to do extra work, in addition to that required by their teacher? *Yes; No*

If yes:

	My child is not concerned by this subject	Never	Very rarely	1-3 times a month	Once a week	2-4 times per week	Almost every day	Every day
Writing								
Spelling								
Reading								
Mathematics - Arithmetic								
Mathematics - Geometry								
Science								
History / Geography								
Music								
Arts								
Sports								

And:

What are you looking for with this extra work? *To help my child review concepts learned with the teacher; To get ahead of the curriculum, to teach new concepts; To help my child overcome certain difficulties; To compensate for the low amount of work proposed by the teacher*

• Have you changed the amount of extra work required of your child during the school vacation period? *No, I kept the same rhythm; Yes, I increased the amount of work required; Yes, I decreased the amount of work required; Yes, I stopped asking my child to do extra work*

• Are you concerned about the impact of the current situation on your child's education? *Yes, a lot; Yes, a little; No, not at all; No opinion*

If yes:

What concerns you most about the impact on your child's education? *Falling behind in the school program; Your child no longer seeing classmates; Your child no longer seeing the teacher; Loss of reference points due to school closures*

• Given the confinement and homeschooling situation, a delay in the school program is a possible consequence. Are there any subjects that you are particularly concerned about?

	My child is not concerned by this subject	Not at all concerned	Not concerned	Concerned	Very concerned	Extremely concerned
Writing						
Spelling						
Reading						
Mathematics - Arithmetic						
Mathematics - Geometry						
Science						
History / Geography						
Music						
Arts						
Sports						

• Is your child with another parent besides you (mother, father) during this lockdown? *Yes; No*

If yes:

Which of these categories best describes the other parent's work situation? *I am a student or in training; I have a full-time job; I have a part-time job; I am currently unemployed*

Which of these categories describes most accurately the other parent's occupation? *Farmer; Craftsman, merchant and company manager; Managers and professionals in higher education Intermediate professions; Employee; Worker; Inactive (never worked); Other*

What is the other parent's net monthly income? *0 € - 999 €;1,000 € - 1,999 €; 2,000 € - 2,999 €; 3,000 € - 3,999 €; 4,000 € - 4,999 €; 5,000 € - 5,999 €;6,000 € - 6,999 €;7,000 € - 7,999 €; 8,000 € - 8,999 €; 9,000 € - 9,999 €; 10,000 € and more*

• A number of phrases that we use to describe ourselves are given below. Read each sentence and choose the answer that best describes how you feel right now. There are no right or wrong answers. Don't spend too much time on any of these and indicate the answer that best describes your current feelings.

	No	Rather no	Rather yes	Yes
I feel calm.				
I feel safe, worry-free, secure.				
I feel tense.				
I feel overworked.				
I feel calm, at ease with myself.				
I feel moved, upset, annoyed.				
The idea of possible problems is worrying me at the moment.				
I feel happy.				
I feel scared.				
I feel at peace with myself.				
I feel confident.				
I feel nervous, irritable.				

I feel scared, frightened.				
I feel indecisive.				
I feel relaxed.				
I am satisfied.				
I am worried, concerned.				
I am confused, I feel bewildered, puzzled.				
I feel solid, calm, level-headed, thoughtful.				
I feel in a good mood, friendly.				

• On a scale of 0 (no stress at all) to 10 (maximum stress imaginable) what is your stress level regarding these 3 different areas since the beginning of the confinement period?

	0	1	2	3	4	5	6	7	8	9	10
Professional stress											
Personal stress											
Overall stress											
