# Supplementary material for: Prior home learning environment is associated with adaptation to homeschooling during COVID lockdown
Source: Heliyon. 2022 Apr 19;8(4):e09294. doi: 10.1016/j.heliyon.2022.e09294 (PMC9017091; doi:10.1016/j.heliyon.2022.e09294)
Supplement: Parental-questionnaire-Covid-Lockdown-T2.pdf [file mmc3.pdf]

Confinement Covid19- Questionnaire Parent

1. Quel est votre lien de parenté avec l'enfant participant à l'étude?

   Une seule réponse possible.

   - Mère
   - Père

2. Votre situation familiale et professionnelle a-t-elle changé depuis votre participation à l'étude? (en dehors de cette période de confinement)

   Une seule réponse possible.

   - Oui ma situation familiale a changé *Passer à la question 6*
   - Oui ma situation professionelle a changé *Passer à la question 3*
   - Oui ma situation familiale ET professionnelle a changé *Passer à la question 7*
   - Non *Passer à la question 11*

Changement de situation professionnelle

3. Laquelle de ces catégories décrit le mieux votre situation professionnelle actuelle ? Une seule réponse possible

   Une seule réponse possible.

   - je suis élève | étudiant(e) | en formation
   - j'ai un emploi à temps plein
   - j'ai un emploi à temps partiel
   - actuellement, je n'ai pas d'emploi

4. Laquelle de ces catégories décrit le mieux votre profession?

Une seule réponse possible.

- Agriculteur
- Artisan, commerçant et chef d'entreprise
- Cadres et professions intellectuelles du supérieur
- Professions intermédiaires
- Employé
- Ouvrier
- Inactif (n'a jamais travaillé)

5. Quel est votre revenu mensuel net? (Merci d'indiquer VOTRE revenu personnel, et non pas celui du foyer)

Une seule réponse possible.

- 0 € - 999 €
- 1,000 € - 1,999 €
- 2,000 € - 2,999 €
- 3,000 € - 3,999 €
- 4,000 € - 4,999 €
- 5,000 € - 5,999 €
- 6,000 € - 6,999 €
- 7,000 € - 7,999 €
- 8,000 € - 8,999 €
- 9,000 € - 9,999 €
- 10,000 € et plus

Changement de situation familiale

6. Ma situation familiale a changé :

Une seule réponse possible.

- Naissance d'un enfant
- Mariage / nouveau concubinage
- Séparation
- Autre

Passer à la question 11

Changement de situation professionnelle et familiale

7. Laquelle de ces catégories décrit le mieux votre situation professionnelle actuelle ? Une seule réponse possible

Une seule réponse possible.

- je suis élève | étudiant(e) | en formation
- j'ai un emploi à temps plein
- j'ai un emploi à temps partiel
- actuellement, je n’ai pas d’emploi

8. Laquelle de ces catégories décrit le mieux votre profession?

Une seule réponse possible.

- Agriculteur
- Artisan, commerçant et chef d'entreprise
- Cadres et professions intellectuelles du supérieur
- Professions intermédiaires
- Employé
- Ouvrier
- Inactif (n'a jamais travaillé)

9. Quel est votre revenu mensuel net? (Merci d'indiquer VOTRE revenu personnel, et non pas celui du foyer)

Une seule réponse possible.

- 0 € - 999 €
- 1,000 € - 1,999 €
- 2,000 € - 2,999 €
- 3,000 € - 3,999 €
- 4,000 € - 4,999 €
- 5,000 € - 5,999 €
- 6,000 € - 6,999 €
- 7,000 € - 7,999 €
- 8,000 € - 8,999 €
- 9,000 € - 9,999 €
- 10,000 € et plus

10. Ma situation familiale a changé :

Une seule réponse possible.

- Naissance d'un enfant
- Mariage / nouveau concubinage
- Séparation
- Autre

Questions confinement

11. Pendant la période de confinement, êtes vous dans un logement où vous avez accès à :

Une seule réponse possible.

- au moins un balcon/une terrasse
- un jardin privatif
- des équipements sportifs
- rien de tout cela

12. Combien de personnes sont confinées à votre domicile (vous inclus)

Une seule réponse possible.

- 1
- 2
- 3
- 4
- 5
- 6
- 7 ou plus

13. De combien de m2 approximativement disposez vous à votre domicile

Une seule réponse possible.

- Moins de 20 m2
- Entre 20 et 40 m2
- Entre 40 et 60m2
- Entre 60 et 80 m2
- Entre 80 et 100 m2
- Plus de 100 m2

14. Est ce que votre enfant inclus dans l'étude dispose d'une chambre individuelle dans votre logemer

Une seule réponse possible.

- Oui
- Non

15. Vous vivez dans :

Une seule réponse possible.

- Une zone urbaine (centre ville, ville)
- Une zone péri-urbaine (banlieu d'une métropole, les environs d'une ville)
- Une zone rurale (campagne)

16. Pendant cette période de confinement, travaillez vous?

Une seule réponse possible.

- oui je suis en télétravail
- oui sur mon site de travail exclusivement
- oui sur mon site de travail et en télétravail
- oui mais mon activité est en partie réduite (chomage partiel)
- non je suis en chômage partiel
- non je suis en arrêt maladie
- non je suis en congés ou en arrêt pour garde d’enfant(s)
- non je suis en recherche d’emploi

17. Cette période de confinement va-t-elle avoir des répercussions sur votre budget ?

Une seule réponse possible.

- très probablement
- probablement
- probablement pas
- certainement pas

18. Cela risque-t-il de vous mettre en situation de précarité ?

Une seule réponse possible.

- très probablement
- probablement
- probablement pas
- certainement pas

19. Hors période de vacances scolaires, durant cette période de confinement, en moyenne sur la semaine, combien de temps par jour consacrez vous à chacune de ces activités ?

Une seule réponse possible par ligne.

	Jamais	Moins de 30 minutes	De 30 minutes à 1 h	De 1h à 2h	De 2h à 3h	De 3h à 4h	De 4h à 5h	De 5h à 6h	Plus 6H
Travailler et/ou étudier									
Prendre soin de vous (se laver, s'habiller, se coiffer, se maquiller...)									
Faire du sport									
S'occuper de son enfant (repas, bain, habillage,...)									
Jouer avec son enfant (jeu de société, jeu vidéo, ...)									
Travailler avec son enfant (école à la maison, devoirs supplémentaires...)									
Faire des activités créatives avec son enfant (musique, dessin...)									

Faire la cuisine, du bricolage, du ménage/rangement									
Regarder les informations									
Lire, regarder la télé (hors info), jouer aux jeux vidéos									
Ruminer des pensées anxieuses									
Echanger sur les réseaux sociaux, téléphoner, envoyer des sms									

Confinement, bien-être et stress

20. Pour chaque proposition, cochez la case qui correspond le mieux à votre vécu depuis le début de période de confinement

Une seule réponse possible par ligne.

	Jamais	Rarement	Parfois	Souvent	Tout le temps
Je me suis senti(e) optimiste quant à l'avenir					
Je me suis senti(e) utile					
Je me suis senti(e) détendu(e)					
Je me suis senti(e) intéressé(e) par les autres					
...					

21. Sur une échelle allant de 0 (absence totale de stess) à 10 (stress maximal imaginable) quel est votr stress concernant ces 3 différents domaines depuis le début de la période de confinement?

Une seule réponse possible par ligne.

	0	1	2	3	4	5	6	7	8	9
Niveau de stress au travail	◯	◯	◯	◯	◯	◯	◯	◯	◯	◯
Niveau de stress personnel	◯	◯	◯	◯	◯	◯	◯	◯	◯	◯
Niveau de stress en général	◯	◯	◯	◯	◯	◯	◯	◯	◯	◯

Confinement et école à la maison

22. Depuis le début du confinement, rencontrez vous des difficultés avec l'école à la maison? pourquc (plusieurs réponses possible)

Plusieurs réponses possibles.

- [ ] Non, aucune difficulté
- [ ] Oui, je manque d'informations de la part de l'enseignant.
- [ ] Oui, je manque d'outils informatiques ou de matiériel (connexion internet, imprimante, livre, ...)
- [ ] Oui, difficulté d'utilisation ou de connexion au site de travail en ligne (ex : Classroom, ecole.cned.fr, ent ...)
- [ ] Oui, quantité de travail ou d'information trop importante
- [ ] Oui, mon enfant a trop de difficultés à suivre/comprendre le contenu proposé ou réaliser les exercices demandés
- [ ] Oui, j'ai des difficultés à comprendre ce qui est demandé ou à accompagner mon enfant
- [ ] Oui, je manque de temps (télétravail/travail, autres enfants à charge, taches domestiques...)

23. Au niveau informatique, votre enfant dispose

Une seule réponse possible.

- d'un ordinateur personnel ou tablette (prêté ou non)
- d'un ordinateur (ou tablette) que vous laissez à sa disposition
- d'un ordinateur (ou tablette) que vous devez également utiliser pour le travail ou qu'au moins un autre enfant doit utiliser pour travailler

24. Pendant ce confinement, hors période de vacances scolaire, combien de temps votre enfant pass t-il chaque jour sur son travail scolaire?

Une seule réponse possible.

- Moins d'une heure
- Entre 1 et 2 heures
- Entre 2 et 3 heures
- Entre 3 et 4 heures
- Entre 4 et 5 heures
- Entre 5 et 6 heures
- Plus de 6 heures

25. Pendant ce confinement, pendant la période de VACANCES scolaire, combien de temps votre enf passe-t-il chaque jour sur son travail scolaire?

Une seule réponse possible.

- Moins d'une heure
- Entre 1 et 2 heures
- Entre 2 et 3 heures
- Entre 3 et 4 heures
- Entre 4 et 5 heures
- Entre 5 et 6 heures
- Plus de 6 heures

Accompagnement de l'école à la maison

26. Pendant ce confinement et hors période de vacances scolaires, à quelle fréquence accompagnez-vous votre enfant dans son travail scolaire demandé par l'enseignant(e) ?

Une seule réponse possible.

- Jamais
- Moins de 15 minutes par jour
- Environ 15-30 minutes par jour
- Entre 30 minutes et 1h par jour
- Entre 1h et 2h par jour
- Entre 2h et 3h par jour
- Entre 3 et 4h par jour
- Entre 4 et 5 h par jour
- Entre 5 et 6 h par jour
- Plus de 6h

27. Pendant ce confinement et hors période de vacances scolaires, quelles(s) est(sont) le(s) matière(s sur laquelle (lesquelles) vous passez le plus de temps avec votre enfant?

Une seule réponse possible par ligne.

	Mon enfant n'est pas concerné par cette matière	Jamais	Très rarement	1-3 fois par mois	1 fois par semaine	2-4 fois par semaine	Quasiment tous les jours	Tous les jours
Français - Ecriture								
Français - Orthographe								
Français - Lecture								
Mathématiques - Arithmétique								
Mathématiques - Géométrie								
Sciences								
Musique								
Histoire-Géographie								
Anglais								
Arts Plastiques								
EPS / Sport								

28. Vous sentez vous capable d’accompagner efficacement votre enfant sur le programme scolaire ?

Une seule réponse possible.

- Oui
- Ce n'est pas facile mais j'essaie au mieux
- Non

29. Considérez vous que la quantité de travail demandée à votre enfant est

Une seule réponse possible.

- Beaucoup trop importante
- Trop importante
- Tout à fait adapté
- Trop faible
- Beaucoup trop faible

30. Pendant ce confinement, comment avez vous organisé la période de vacances scolaires?

Une seule réponse possible.

- En essayant de la faire ressembler au mieux aux vacances "habituelles" : pas d'école du tout
- En relachant le ryhtme des semaines précédentes mais en poursuivant certaines activités scolaires à la maison malgré tout
- En maintenant le rythme scolaire des semaines précédentes

31. Vous arrive-t-il de demander à votre enfant du travail supplémentaire, en plus de celui demandé p son enseignant?

Une seule réponse possible.

- Oui
- Non

Travail supplémentaire

32. Pendant ce confinement et hors période de vacances scolaires, dans quelle(s) matière(s) avez vou demandé du travail supplémentaire à votre enfant? A quelle fréquence?

Une seule réponse possible par ligne.

	Mon enfant n'est pas concerné par cette matière	Jamais	Très rarement	1-3 fois par mois	1 fois par semaine	2-4 fois par semaine	Quasiment tous les jours	Tous les jours
Français - Ecriture	⬭	⬭	⬭	⬭	⬭	⬭	⬭	⬭
Français - Orthographe	⬭	⬭	⬭	⬭	⬭	⬭	⬭	⬭
Français - Lecture	⬭	⬭	⬭	⬭	⬭	⬭	⬭	⬭
Mathématiques - Arithmétique	⬭	⬭	⬭	⬭	⬭	⬭	⬭	⬭
Mathématiques - Géométrie	⬭	⬭	⬭	⬭	⬭	⬭	⬭	⬭
Sciences	⬭	⬭	⬭	⬭	⬭	⬭	⬭	⬭
Musique	⬭	⬭	⬭	⬭	⬭	⬭	⬭	⬭
Histoire-Géographie	⬭	⬭	⬭	⬭	⬭	⬭	⬭	⬭
Anglais	⬭	⬭	⬭	⬭	⬭	⬭	⬭	⬭
Arts Plastiques	⬭	⬭	⬭	⬭	⬭	⬭	⬭	⬭
EPS / Sport	⬭	⬭	⬭	⬭	⬭	⬭	⬭	⬭

33. Que recherchez-vous avec ce travail supplémentaire? (plusieurs réponses possibles)

Plusieurs réponses possibles.

- ☐ Faire réviser mon enfant des notions apprises avec l'enseignant(e)
- ☐ Prendre de l'avance sur le programme, enseigner de nouvelles notions
- ☐ Aider mon enfant à surmonter certaines difficultés
- ☐ Compenser la trop faible quantité de travail proposé par l'enseignant(e)

34. Avez vous changé le rythme de travail supplémentaire demandé à votre enfant pendant la période de vacances scolaires?

Plusieurs réponses possibles.

- [ ] Non j'ai maintenu un rythme identique
- [ ] Oui j'ai augmenté la quantité de travail demandé
- [ ] Oui j'ai diminué la quantité de travail demandé
- [ ] Oui j'ai cessé de demandér du travail supplémentaire à mon enfant

Ecole à la maison et attente

35. Etes vous préoccupé par les conséquences de la situation actuelle sur la scolarité de votre enfant?

Une seule réponse possible.

- ( ) Oui, beaucoup
- ( ) Oui, un peu
- ( ) Non, pas du tout *Passer à la question 37*
- ( ) Pas d'opinion *Passer à la question 37*

36. Q'est ce qui vous préoccupe le plus concernant les conséquences sur la scolarité de votre enfant?

Une seule réponse possible.

- ( ) Le retard pris sur le programme scolaire
- ( ) Le fait que votre enfant ne voit plus ses camarades de classe
- ( ) Le fait que votre enfant ne voit plus son enseignant
- ( ) Une perte de repères suite à la fermeture des écoles

Retard sur le programme scolaire

37. Etant donné la situation de confinement et d'école à la maison, un retard sur le programme scolair est une conséquence possible. Y a-t-il des disciplines pour lesquelles cela vous inquiète plus particulièrement?

Une seule réponse possible par ligne.

	Mon enfant n'est pas concerné par cette matière	Pas du tout inquiet(e)	Pas inquiet(e)	Inquiet(e)	Très inquiet(e)	Extrêmemer inquiet(e)
Français - Ecriture						
Français - Orthographe						
Français - Lecture						
Mathématiques - Arithmétique						
Mathématiques - Géométrie						
Sciences						
Musique						
Histoire-Géographie						
Anglais						
Arts Plastiques						
EPS / Sport						

38. Votre enfant est-il confiné avec un autre parent en plus de vous (père ou mère)?

Une seule réponse possible.

- Oui
- Non *Passer à la section 14 (Merci pour votre participation!).*

Situation professionnelle de l'autre parent

39. Laquelle de ces catégories décrit le mieux la situation professionnelle actuelle de l'autre parent? U seule réponse possible

Une seule réponse possible.

- je suis élève | étudiant(e) | en formation
- j'ai un emploi à temps plein
- j'ai un emploi à temps partiel
- actuellement, je n’ai pas d’emploi

40. Laquelle de ces catégories décrit le mieux la profession de l'autre parent?

Une seule réponse possible.

- Agriculteur
- Artisan, commerçant et chef d'entreprise
- Cadres et professions intellectuelles du supérieur
- Professions intermédiaires
- Employé
- Ouvrier
- Inactif (n'a jamais travaillé)

41. Quel est le revenu mensuel net de l'autre parent?

Une seule réponse possible.

- 0 € - 999 €
- 1,000 € - 1,999 €
- 2,000 € - 2,999 €
- 3,000 € - 3,999 €
- 4,000 € - 4,999 €
- 5,000 € - 5,999 €
- 6,000 € - 6,999 €
- 7,000 € - 7,999 €
- 8,000 € - 8,999 €
- 9,000 € - 9,999 €
- 10,000 € et plus

42. Pendant cette période de confinement, travaille-t-il/elle?

Une seule réponse possible.

- Oui en télétravail
- Oui sur son site de travail exclusivement
- Oui sur son site de travail et en télétravail
- Oui mais son activité est en partie réduite chômage partiel)
- Non, en chômage partiel
- Non, en arrêt maladie
- Non, en congés ou en arrêt pour garde d'enfant(s)
- Non, en recherche d'emploi

Passer à la question 11

Merci pour votre participation!

Ce contenu n'est ni rédigé, ni cautionné par Google.

Google Forms
