# Supplementary material for: Prior home learning environment is associated with adaptation to homeschooling during COVID lockdown
Source: Heliyon. 2022 Apr 19;8(4):e09294. doi: 10.1016/j.heliyon.2022.e09294 (PMC9017091; doi:10.1016/j.heliyon.2022.e09294)
Supplement: Parental-questionnaire-T1.pdf [file mmc4.pdf]

Informations sur le milieu familial (8 ans)

*Obligatoire

1. **Merci d'entrer ici le code d'anonymat qui vous a été remis (exemple : P 00X ou M 00X)** *

2. **Quel est votre lien de parenté avec l'enfant participant à l'étude?** *
   Plusieurs réponses possibles.
   - [ ] Mère
   - [ ] Père
   - [ ] Autre : ____________

3. **Quel est votre code postal?** *

4. **Quelle est votre année de naissance?** *

5. **Quel est votre lieu de naissance? (merci de préciser le nom de la ville et le pays de naissance)** *

6. **Quel est le plus haut niveau d'étude que vous avez atteint?** *
Une seule réponse possible.

- Ecole primaire ou niveau 6e, 5e,4e, 3e
- Diplôme national du brevet (DNB) ou Brevet d'études du premier cycle (BEPC)
- BEP, CAP ou fin 2nde ou 1ère
- Niveau fin terminale
- Baccalauréat général ou professionnel ou équivalence (BTA, BTI)
- BTS, DUT
- DEUG, DEUST (L2)
- Licence (L3)
- Maitrise (M1)
- Master (M2), DESS, DEA
- Diplôme Grande Ecole
- Diplôme de Docteur en Santé (Médecine, Pharmacie...)
- Doctorat (sauf Santé)

7. **Laquelle de ces catégories décrit le mieux votre situation professionnelle actuelle ? Une seule réponse possible** *
Une seule réponse possible.

- je suis élève | étudiant(e) | en formation
- j'ai un emploi à temps plein
- j'ai un emploi à temps partiel
- actuellement, je n'ai pas d'emploi

8. **Laquelle de ces catégories décrit le mieux votre profession?** *
Une seule réponse possible.

- Agriculteur
- Artisan, commerçant et chef d'entreprise
- Cadres et professions intellectuelles du supérieur
- Professions intermédiaires
- Employé
- Ouvrier
- Inactif (n'a jamais travaillé)
- Autre : ____________________

9. **Quel est votre revenu mensuel net? (Merci d'indiquer VOTRE revenu personnel, et non pas celui du foyer)** *

Une seule réponse possible.

- 0 € - 999 €
- 1,000 € - 1,999 €
- 2,000 € - 2,999 €
- 3,000 € - 3,999 €
- 4,000 € - 4,999 €
- 5,000 € - 5,999 €
- 6,000 € - 6,999 €
- 7,000 € - 7,999 €
- 8,000 € - 8,999 €
- 9,000 € - 9,999 €
- 10,000 € et plus

10. **Quelles sont les langues parlées à la maison?** *

____

11. **Avez vous déjà vous-même reçu le diagnostic d'un trouble de l'apprentissage (un trouble VOUS concernant)?** *

Une seule réponse possible.

- Oui
- Non *Passez à la question 17.*

Diagnostic d'un trouble de l'apprentissage

12. **Lequel (ou lesquels)?** *

Plusieurs réponses possibles.

- [ ] Dsylexie (trouble de l'apprentissage de la lecture)
- [ ] Dyscalculie (trouble de l'apprentissage des mathématiques)
- [ ] Dyspraxie (trouble du développement moteur et de l'écriture)
- [ ] Dysphasie (trouble du langage oral)
- [ ] TDA/H (trouble de l'attention)
- [ ] Autre : ______________________

13. **A quel âge avez vous été diagnostiqué?** *

____

14. **Un autre membre de votre famille (autre que l'enfant qui participe à l'étude) a-t- il déjà été diagnostiqué avec un trouble de l'apprentissage?** *

Une seule réponse possible.

- Oui
- Non *Passez à la question 17.*

Diagnostic d'un trouble de l'apprentissage

15. **Lequel (ou lesquels)?** *

Plusieurs réponses possibles.

- [ ] Dsylexie (trouble de l'apprentissage de la lecture)
- [ ] Dyscalculie (trouble de l'apprentissage des mathématiques)
- [ ] Dyspraxie (trouble du développement moteur et de l'écriture)
- [ ] Dysphasie (trouble du langage oral)
- [ ] TDA/H (trouble de l'attention)
- [ ] Autre : ____________________

16. **A quel âge ce membre de la famille a-t-il été diagnostiqué?** *

____

17. **Lire les affirmations suivantes. S'il vous plait, en utilisant l'échelle proposée, indiquez à quel point vous êtes d'accord avec les affirmations en cochant la case appropriée.** *

Une seule réponse possible par ligne.

	Ne sait pas	Pas du tout d'accord	Pas d'accord	Assez d'accord	Tout à fait d'accord
Quand j'étais à l'école, j'étais bon en mathématiques.					
Quand j'étais à l'école, j'étais bon en activités liées à la maitrise du langage, comme la lecture par exemple.					
Mon emploi implique beaucoup de rapports écrits.					
Mon emploi implique l'utilisation des mathématiques.					
Je trouve les mathématiques agréables.					
Je trouve l'écriture agréable.					
Je trouve la lecture agréable.					
J'évite des situations impliquant l'écriture					
J'évite des situations impliquant la lecture					
J'évite des situations impliquant les mathématiques.					

Informations socio démographiques

18. **Actuellement, EN PLUS DE VOUS-MÊME, combien d'adultes vivent dans votre logement? (Âge supérieur à 18 ans)** *

Une seule réponse possible.

- 0 *Passez à la question 39.*
- 1 *Passez à la question 19.*
- 2 *Passez à la question 21.*
- 3 *Passez à la question 25.*
- 4 *Passez à la question 31.*

Informations sur l'adulte supplémentaire dans le logement

19. **Quel est votre lien de parenté avec l'autre adulte?** *

20. **Quel est l'âge de l'autre adulte?** *

Passez à la question 39.

Informations pour deux adultes supplémentaires dans le logement

21. **Quel est votre lien de parenté avec le premier adulte?** *

22. **Quel est l'âge du premier adulte?** *

23. **Quel est votre lien de parenté avec le deuxième adulte?** *

24. **Quel est l'âge du deuxième adulte?** *

Passez à la question 39.

Informations pour trois adultes supplémentaires dans le logement

25. **Quel est votre lien de parenté avec le premier adulte?** *

26. **Quel est l'âge du premier adulte?** *

27. **Quel est votre lien de parenté avec le deuxième adulte?** *

28. **Quel est l'âge du deuxième adulte?** *

29. **Quel est votre lien de parenté avec le troisième adulte?** *

30. **Quel est l'âge du troisième adulte?** *

Passez à la question 39.

Informations pour quatre adultes supplémentaires dans le logement

31. **Quel est votre lien de parenté avec le premier adulte?** *

32. **Quel est l'âge du premier adulte?** *

33. **Quel est votre lien de parenté avec le deuxième adulte?** *

34. **Quel est l'âge du deuxième adulte?** *

35. **Quel est votre lien de parenté avec le troisième adulte?** *

36. **Quel est l'âge du troisième adulte?** *

37. **Quel est votre lien de parenté avec le quatrième adulte?** *

38. **Quel est l'âge du quatrième adulte?** *

Passez à la question 39.

39. **Actuellement, en plus de votre enfant concerné par l'étude, combien d'enfants vivent dans votre logement? (Âge inférieur à 18 ans)** *
Une seule réponse possible.

- 0 *Passez à la question 112.*
- 1 *Passez à la question 40.*
- 2 *Passez à la question 42.*
- 3 *Passez à la question 46.*
- 4 *Passez à la question 52.*
- 5 *Passez à la question 60.*
- 6 *Passez à la question 70.*
- 7 *Passez à la question 82.*
- 8 *Passez à la question 96.*

Informations pour un enfant (autre que celui participant à l'étude)

40. **Quel est votre lien de parenté avec le premier enfant?** *

41. **Quel est l'âge du premier enfant?** *

Passez à la question 112.

Informations pour deux enfants (autre que celui participant à l'étude)

42. **Quel est votre lien de parenté avec le premier enfant?** *

43. **Quel est l'âge du premier enfant?** *

44. **Quel est votre lien de parenté avec le deuxième enfant?** *

45. **Quel est l'âge du deuxième enfant?** *

Passez à la question 112.

Informations pour trois enfants (autre que celui participant à l'étude)

46. **Quel est votre lien de parenté avec le premier enfant?** *

47. **Quel est l'âge du premier enfant?** *

48. **Quel est votre lien de parenté avec le deuxième enfant?** *

49. **Quel est l'âge du deuxième enfant?** *

50. **Quel est votre lien de parenté avec le troisième enfant?** *

51. **Quel est l'âge du troisième enfant?** *

Passez à la question 112.

Informations pour quatre enfants (autre que celui participant à l'étude)

52. **Quel est votre lien de parenté avec le premier enfant?** *

53. **Quel âge du premier enfant?** *

54. **Quel est votre lien de parenté avec le deuxième enfant?** *

55. **Quel âge du deuxième enfant?** *

56. **Quel est votre lien de parenté avec le troisième enfant?** *

57. **Quel est l'âge du troisième enfant?** *

58. **Quel est votre lien de parenté avec le quatrième enfant?** *

59. **Quel est l'âge du quatrième enfant?** *

Passez à la question 112.

Informations pour cinq enfants (autre que celui participant à l'étude)

60. **Quel est votre lien de parenté avec le premier enfant?** *

61. **Quel âge du premier enfant?** *

62. **Quel est votre lien de parenté avec le deuxième enfant?** *

63. **Quel âge du deuxième enfant?** *

64. **Quel est votre lien de parenté avec le troisième enfant? ***

65. **Quel est l'âge du troisième enfant? ***

66. **Quel est votre lien de parenté avec le quatrième enfant? ***

67. **Quel est l'âge du quatrième enfant? ***

68. **Quel est votre lien de parenté avec le cinquième enfant? ***

69. **Quel est l'âge du cinquième enfant? ***

Passez à la question 112.

Informations pour six enfants (autre que celui participant à l'étude)

70. **Quel est votre lien de parenté avec le premier enfant? ***

71. **Quel âge du premier enfant? ***

72. **Quel est votre lien de parenté avec le deuxième enfant? ***

73. **Quel âge du deuxième enfant? ***

74. **Quel est votre lien de parenté avec le troisième enfant?** *

75. **Quel est l'âge du troisième enfant?** *

76. **Quel est votre lien de parenté avec le quatrième enfant?** *

77. **Quel est l'âge du quatrième enfant?** *

78. **Quel est votre lien de parenté avec le cinquième enfant?** *

79. **Quel est l'âge du cinquième enfant?** *

80. **Quel est votre lien de parenté avec le sixième enfant?** *

81. **Quel est l'âge du sixième enfant?** *

Passez à la question 112.

Informations pour sept enfants (autre que celui participant à l'étude)

82. **Quel est votre lien de parenté avec le premier enfant?** *

83. **Quel âge du premier enfant?** *

84. **Quel est votre lien de parenté avec le deuxième enfant? ***

85. **Quel âge du deuxième enfant? ***

86. **Quel est votre lien de parenté avec le troisième enfant? ***

87. **Quel est l'âge du troisième enfant? ***

88. **Quel est votre lien de parenté avec le quatrième enfant? ***

89. **Quel est l'âge du quatrième enfant? ***

90. **Quel est votre lien de parenté avec le cinquième enfant? ***

91. **Quel est l'âge du cinquième enfant? ***

92. **Quel est votre lien de parenté avec le sixième enfant? ***

93. **Quel est l'âge du sixième enfant? ***

94. **Quel est votre lien de parenté avec le septième enfant? ***

95. **Quel est l'âge du septième enfant? ***

Passez à la question 112.

Informations pour huit enfants (autre que celui participant à l'étude)

96. **Quel est votre lien de parenté avec le premier enfant?** *

97. **Quel âge du premier enfant?** *

98. **Quel est votre lien de parenté avec le deuxième enfant?** *

99. **Quel âge du deuxième enfant?** *

100. **Quel est votre lien de parenté avec le troisième enfant?** *

101. **Quel est l'âge du troisième enfant?** *

102. **Quel est votre lien de parenté avec le quatrième enfant?** *

103. **Quel est l'âge du quatrième enfant?** *

104. **Quel est votre lien de parenté avec le cinquième enfant?** *

105. **Quel est l'âge du cinquième enfant?** *

106. **Quel est votre lien de parenté avec le sixième enfant?** *

107. **Quel est l'âge du sixième enfant?** *

108. **Quel est votre lien de parenté avec le septième enfant?** *

109. **Quel est l'âge du septième enfant?** *

110. **Quel est votre lien de parenté avec le huitième enfant?** *

111. **Quel est l'âge du huitième enfant?** *

112. **Lire les affirmations suivantes. S'il vous plait, en utilisant l'échelle proposée, indiquer à quel point vous êtes d'accord ou non.** *
Une seule réponse possible par ligne.

	Ne sait pas	Pas du tout d'accord	Pas d'accord	Assez d'accord	Tout à fait d'accord
Je suis sûr/e de savoir comment impliquer mon enfant dans une activité mathématique.					
Je suis sûr/e de savoir comment impliquer mon enfant dans une activité de lecture.					
Chez moi, les compétences mathématiques sont enseignées au hasard des occasions de la journée.					
Chez moi, les compétences en lecture sont enseignées au hasard des occasions de la journée.					

Repères à acquérir avant la FIN de l'école primaire

Selon vous, à quel point est-ce important pour votre enfant d’acquérir les repères suivants avant la FIN de l'école primaire?

113. **Savoir communiquer correctement avec des adultes (hors famille)** *

Une seule réponse possible.

- Pas d'opinion
- Pas du tout important
- Pas important
- Important
- Très important
- Extrêmement important

114. **Construire et entretenir des liens d'amitié avec des enfants du même âge** *

Une seule réponse possible.

- Pas d'opinion
- Pas du tout important
- Pas important
- Important
- Très important
- Extrêmement important

115. **Appliquer les consignes, respecter les règles de vie collective** *

Une seule réponse possible.

- Pas d'opinion
- Pas du tout important
- Pas important
- Important
- Très important
- Extrêmement important

Repères à acquérir avant la FIN de l'école primaire

Selon vous, à quel point est-ce important pour votre enfant d’acquérir les repères suivants avant la FIN de l'école primaire?

116. **Connaitre et localiser de grands repères géographiques sur des supports cartographiques variés** *

Une seule réponse possible.

- Pas d'opinion
- Pas du tout important
- Pas important
- Important
- Très important
- Extrêmement important

117. **Identifier quelques enjeux du développement durable** *

Une seule réponse possible.

- Pas d'opinion
- Pas du tout important
- Pas important
- Important
- Très important
- Extrêmement important

118. **Connaitre et situer dans le temps de grandes périodes historiques** *

Une seule réponse possible.

- Pas d'opinion
- Pas du tout important
- Pas important
- Important
- Très important
- Extrêmement important

Repères à acquérir avant la FIN de l'école primaire

Selon vous, à quel point est-ce important pour votre enfant d'acquérir les repères suivants avant la FIN de l'école primaire?

119. **Compter jusqu'à 100** *

Une seule réponse possible.

- Pas d'opinion *Passez à la question 121.*
- Pas du tout important *Passez à la question 121.*
- Pas important *Passez à la question 121.*
- Important *Passez à la question 120.*
- Très important *Passez à la question 120.*
- Extrêmement important *Passez à la question 120.*

Repères à acquérir avant la FIN de l'école primaire

Selon vous, à quel point est-ce important pour votre enfant d’acquérir les repères suivants avant la FIN de l'école primaire?

120. **Compter jusqu'à 1000** *

Une seule réponse possible.

- Pas d'opinion
- Pas du tout important
- Pas important
- Important
- Très important
- Extrêmement important

Repères à acquérir avant la FIN de l'école primaire

Selon vous, à quel point est-ce important pour votre enfant d’acquérir les repères suivants avant la FIN de l'école primaire?

121. **Lire les nombres écrits jusqu'à 100 (1, 2, 3, ...)** *

Une seule réponse possible.

- Pas d'opinion *Passez à la question 123.*
- Pas du tout important *Passez à la question 123.*
- Pas important *Passez à la question 123.*
- Important
- Très important
- Extrêmement important

Repères à acquérir avant la FIN de l'école primaire

Selon vous, à quel point est-ce important pour votre enfant d’acquérir les repères suivants avant la FIN de l'école primaire?

122. **Lire les nombres écrits jusqu'à 1000 (1, 2, 3, ...)** *

Une seule réponse possible.

- Pas d'opinion *Passez à la question 123.*
- Pas du tout important *Passez à la question 123.*
- Pas important *Passez à la question 123.*
- Important
- Très important
- Extrêmement important

Repères à acquérir avant la FIN de l'école primaire

Selon vous, à quel point est-ce important pour votre enfant d’acquérir les repères suivants avant la FIN de l'école primaire?

123. **Savoir faire des sommes simples sans compter sur ses doigts (ex : 2+2)** *

Une seule réponse possible.

- Pas d'opinion *Passez à la question 132.*
- Pas du tout important *Passez à la question 132.*
- Pas important *Passez à la question 132.*
- Important
- Très important
- Extrêmement important

Repères à acquérir avant la FIN de l'école primaire

Selon vous, à quel point est-ce important pour votre enfant d’acquérir les repères suivants avant la FIN de l'école primaire?

124. **Savoir faire des additions complexes (ex 15+121 ; 11+447)** *

Une seule réponse possible.

- Pas d'opinion *Passez à la question 129.*
- Pas du tout important *Passez à la question 129.*
- Pas important *Passez à la question 129.*
- Important *Passez à la question 125.*
- Très important *Passez à la question 125.*
- Extrêmement important *Passez à la question 125.*

Repères à acquérir avant la FIN de l'école primaire

Selon vous, à quel point est-ce important pour votre enfant d’acquérir les repères suivants avant la FIN de l'école primaire?

125. **Savoir faire des multiplications simples (ex : 2x6)** *

Une seule réponse possible.

- Pas d'opinion *Passez à la question 127.*
- Pas du tout important *Passez à la question 127.*
- Pas important *Passez à la question 127.*
- Important
- Très important
- Extrêmement important

Repères à acquérir avant la FIN de l'école primaire

Selon vous, à quel point est-ce important pour votre enfant d’acquérir les repères suivants avant la FIN de l'école primaire?

126. **Savoir faire des multiplications plus complexes (ex : 14x7)** *

Une seule réponse possible.

- Pas d'opinion
- Pas du tout important
- Pas important
- Important
- Très important
- Extrêmement important

Repères à acquérir avant la FIN de l'école primaire

Selon vous, à quel point est-ce important pour votre enfant d'acquérir les repères suivants avant la FIN de l'école primaire?

127. **Connaître les fractions et savoir les utiliser (ex : 5/6)** *

Une seule réponse possible.

- Pas d'opinion
- Pas du tout important
- Pas important
- Important
- Très important
- Extrêmement important

128. **Savoir faire des divisions (ex : 30÷5)** *

Une seule réponse possible.

- Pas d'opinion
- Pas du tout important
- Pas important
- Important
- Très important
- Extrêmement important

Repères à acquérir avant la FIN de l'école primaire

Selon vous, à quel point est-ce important pour votre enfant d'acquérir les repères suivants avant la FIN de l'école primaire?

129. **Connaitre les nombres décimaux (ex : 3,2)** *

Une seule réponse possible.

- Pas d'opinion *Passez à la question 131.*
- Pas du tout important *Passez à la question 131.*
- Pas important *Passez à la question 131.*
- Important
- Très important
- Extrêmement important

Repères à acquérir avant la FIN de l'école primaire

Selon vous, à quel point est-ce important pour votre enfant d'acquérir les repères suivants avant la FIN de l'école primaire?

130. **Calculer avec des nombres décimaux** *

Une seule réponse possible.

- Pas d'opinion
- Pas du tout important
- Pas important
- Important
- Très important
- Extrêmement important

Repères à acquérir avant la FIN de l'école primaire

Selon vous, à quel point est-ce important pour votre enfant d'acquérir les repères suivants avant la FIN de l'école primaire?

131. **Connaitre les probabilités et les utiliser** *

Une seule réponse possible.

- Pas d'opinion
- Pas du tout important
- Pas important
- Important
- Très important
- Extrêmement important

Repères à acquérir avant la FIN de l'école primaire

Selon vous, à quel point est-ce important pour votre enfant d'acquérir les repères suivants avant la FIN de l'école primaire?

132. **Reconnaître, nommer et reproduire des figures géométriques** *

Une seule réponse possible.

- Pas d'opinion *Passez à la question 134.*
- Pas du tout important *Passez à la question 134.*
- Pas important *Passez à la question 134.*
- Important
- Très important
- Extrêmement important

Repères à acquérir avant la FIN de l'école primaire

Selon vous, à quel point est-ce important pour votre enfant d'acquérir les repères suivants avant la FIN de l'école primaire?

133. **Reconnaître et utiliser quelques notions de géométrie (ex : notions de perpendicularité ou de parallélisme)** *

Une seule réponse possible.

- Pas d'opinion
- Pas du tout important
- Pas important
- Important
- Très important
- Extrêmement important

Repères à acquérir avant la FIN de l'école primaire

Selon vous, à quel point est-ce important pour votre enfant d'acquérir les repères suivants avant la FIN de l'école primaire?

134. **Savoir utiliser une règle pour mesurer une longueur** *

Une seule réponse possible.

- Pas d'opinion
- Pas du tout important
- Pas important
- Important
- Très important
- Extrêmement important

135. **Exprimer une grandeur mesurée ou calculée dans une unité appropriée** *

Une seule réponse possible.

- Pas d'opinion
- Pas du tout important
- Pas important
- Important
- Très important
- Extrêmement important

Repères à acquérir avant la FIN de l'école primaire

Selon vous, à quel point est-ce important pour votre enfant d'acquérir les repères suivants avant la FIN de l'école primaire?

136. **Ecrire à la main de manière fluide et efficace** *

Une seule réponse possible.

- Pas d'opinion *Passez à la question 139.*
- Pas du tout important *Passez à la question 139.*
- Pas important *Passez à la question 139.*
- Important
- Très important
- Extrêmement important

Repères à acquérir avant la FIN de l'école primaire

Selon vous, à quel point est-ce important pour votre enfant d'acquérir les repères suivants avant la FIN de l'école primaire?

137. **Rédiger un texte d'environ une demi-page** *

Une seule réponse possible.

- Pas d'opinion
- Pas du tout important
- Pas important
- Important
- Très important
- Extrêmement important

138. **Recourir à l'écriture de manière autonome (ex : pour garder des traces de ses lectures)** *

Une seule réponse possible.

- Pas d'opinion
- Pas du tout important
- Pas important
- Important *Passez à la question 139.*
- Très important *Passez à la question 139.*
- Extrêmement important *Passez à la question 139.*

Repères à acquérir avant la FIN de l'école primaire

Selon vous, à quel point est-ce important pour votre enfant d'acquérir les repères suivants avant la FIN de l'école primaire?

139. **Savoir orthographier les mots fréquents** *

Une seule réponse possible.

- Pas d'opinion *Passez à la question 141.*
- Pas du tout important *Passez à la question 141.*
- Pas important *Passez à la question 141.*
- Important *Passez à la question 140.*
- Très important *Passez à la question 140.*
- Extrêmement important *Passez à la question 140.*

Repères à acquérir avant la FIN de l'école primaire

Selon vous, à quel point est-ce important pour votre enfant d'acquérir les repères suivants avant la FIN de l'école primaire?

140. **Savoir orthographier les mots invariables mémorisés (ex : puis, dedans, enfin...)** *

Une seule réponse possible.

- Pas d'opinion
- Pas du tout important
- Pas important
- Important
- Très important
- Extrêmement important

Repères à acquérir avant la FIN de l'école primaire

Selon vous, à quel point est-ce important pour votre enfant d'acquérir les repères suivants avant la FIN de l'école primaire?

141. **Lire des livres imagés simples (=des livres avec des images et du texte)** *

Une seule réponse possible.

- Pas d'opinion *Passez à la question 146.*
- Pas du tout important *Passez à la question 146.*
- Pas important *Passez à la question 146.*
- Important *Passez à la question 142.*
- Très important *Passez à la question 142.*
- Extrêmement important *Passez à la question 142.*

Repères à acquérir avant la FIN de l'école primaire

Selon vous, à quel point est-ce important pour votre enfant d'acquérir les repères suivants avant la FIN de l'école primaire?

142. **Lire et comprendre des petits paragraphes** *

Une seule réponse possible.

- Pas d'opinion
- Pas du tout important
- Pas important
- Important
- Très important
- Extrêmement important

143. **Lire des chapitres de livres (des livres avec uniquement du texte)** *

Une seule réponse possible.

- Pas d'opinion *Passez à la question 146.*
- Pas du tout important *Passez à la question 146.*
- Pas important *Passez à la question 146.*
- Important *Passez à la question 144.*
- Très important *Passez à la question 144.*
- Extrêmement important *Passez à la question 144.*

Repères à acquérir avant la FIN de l'école primaire

Selon vous, à quel point est-ce important pour votre enfant d'acquérir les repères suivants avant la FIN de l'école primaire?

144. **Lire un livre pour enfant en intégralité** *

Une seule réponse possible.

- Pas d'opinion
- Pas du tout important
- Pas important
- Important
- Très important
- Extrêmement important

145. **Comprendre et interpréter des textes ou des œuvres** *

Une seule réponse possible.

- Pas d'opinion
- Pas du tout important
- Pas important
- Important
- Très important
- Extrêmement important

Repères à acquérir avant la FIN de l'école primaire

Selon vous, à quel point est-ce important pour votre enfant d'acquérir les repères suivants avant la FIN de l'école primaire?

146. **Réaliser une présentation orale, un compte rendu d'activité (ex : exposé)** *

Une seule réponse possible.

- Pas d'opinion
- Pas du tout important
- Pas important
- Important *Après avoir répondu à la dernière question de cette section, passez à la question 148.*
- Très important *Après avoir répondu à la dernière question de cette section, passez à la question 148.*
- Extrêmement important *Après avoir répondu à la dernière question de cette section, passez à la question 148.*

147. **Participer à des échanges oraux (ex : débat)** *

Une seule réponse possible.

- Pas d'opinion
- Pas du tout important
- Pas important
- Important *Passez à la question 148.*
- Très important *Passez à la question 148.*
- Extrêmement important *Passez à la question 148.*

Repères à acquérir avant la FIN de l'école primaire

Selon vous, à quel point est-ce important pour votre enfant d'acquérir les repères suivants avant la FIN de l'école primaire?

148. **Mettre en oeuvre un protocole expérimental, concevoir ou produire tout ou partie d'un objet technique.** *

Une seule réponse possible.

- Pas d'opinion
- Pas du tout important
- Pas important
- Important
- Très important
- Extrêmement important

149. **Pratiquer diverses formes de créations littéraires et artistiques** *

Une seule réponse possible.

- Pas d'opinion
- Pas du tout important
- Pas important
- Important
- Très important
- Extrêmement important

Repères à acquérir avant la FIN de l'école primaire

Selon vous, à quel point est-ce important pour votre enfant d'acquérir les repères suivants avant la FIN de l'école primaire?

150. **Réussir des performances sportives (course, saut, ...)** *

Une seule réponse possible.

- Pas d'opinion
- Pas du tout important
- Pas important
- Important
- Très important
- Extrêmement important

151. **Comprendre le fonctionnement général du corps humain** *

Une seule réponse possible.

- Pas d'opinion
- Pas du tout important
- Pas important
- Important
- Très important
- Extrêmement important

Scolarité

152. **Selon vous, comment évalueriez-vous les capacités de votre enfant dans les domaines suivants ?** *

Une seule réponse possible par ligne.

	Pas d'opinion	Grandes difficultés	Difficultés	Capacités dans la moyenne	Très bonnes capacités	Capacités extrêmement bonnes
Ecriture						
Orthographe						
Lecture						
Mathématiques						
Sciences						
Histoire/ géographie						
Musique						
Arts plastiques						
EPS (éducation physique et sportive)						

Scolarité

153. **Selon vous à quel point la réussite scolaire dans ces différents domaines est importante ?** *

Une seule réponse possible par ligne.

	Pas d'opinion	Pas du tout important	Pas important	Important	Très important	Extrêmement important
Ecriture						
Orthographe						
Lecture						
Mathématiques						
Sciences						
Histoire/ géographie						
Musique						
Arts plastiques						
EPS (éducation physique et sportive)						

Scolarité

154. **A quelle fréquence demandez-vous à votre enfant de réaliser des exercices/activités dans les domaines suivants (EN DEHORS des devoirs donnés par l'école) ?** *

Une seule réponse possible par ligne.

	Jamais	Moins de 15 minutes par jour	Environ 15-30 minutes par jour	Environ 30 min -1h par jour	Plus d'1h par jour
Mathématiques (ex : calcul mental)					
Lecture (ex : lecture de livres silencieuse ou à haute voix)					
Pratique de l'orthographe (ex : dictée de mots)					
Ecriture (écrire des lettres, des rédactions; prendre des notes)					
Sciences (ex : planter des graines)					
Musique (ex : pratique d'un instrument)					
Arts plastiques (ex : réalisation de peintures)					
Sport (ex : pratique d'une activité sportive)					

155. **Est-ce que votre enfant a fréquenté l'école maternelle entre les âges de 2 et 3 ans?** *

Une seule réponse possible.

- Oui
- Non *Passez à la question 157.*

156. **En moyenne, combien d'heures par semaine votre enfant a-t-il fréquenté l'école maternelle entre 2 et 3 ans?** *

157. **Au cours d'une semaine normale (hors week end), combien d'heures PAR JOUR en moyenne passez-vous avec votre enfant?** *

Une seule réponse possible.

- Moins d'1 heure
- Entre 1 et 2 heures
- Entre 2 et 3 heures
- Entre 3 et 4 heures
- Entre 4 et 5 heures
- Entre 5 et 6 heures
- Entre 6 et 7 heures
- Plus de 7 heures

Au cours d'une semaine classique, combien d'heures PAR JOUR en moyenne est ce que votre enfant passe à réaliser les activités suivantes?

158. **Regarder des dessins animés ou émissions de TV non éducatives** *

Une seule réponse possible.

- Ne s'applique pas à mon enfant
- Moins d'1 heure
- Entre 1 et 2 heures
- Entre 2 et 3 heures
- Entre 3 et 4 heures
- Plus de 4 heures

159. **Regarder des émissions de TV éducatives** *

Une seule réponse possible.

- Ne s'applique pas à mon enfant
- Moins d'1 heure
- Entre 1 et 2 heures
- Entre 2 et 3 heures
- Entre 3 et 4 heures
- Plus de 4 heures

160. **Jouer à des jeux vidéos non éducatifs (tablette, ordinateur, console...)** *

Une seule réponse possible.

- Ne s'applique pas à mon enfant
- Moins d'1 heure
- Entre 1 et 2 heures
- Entre 2 et 3 heures
- Entre 3 et 4 heures
- Plus de 4 heures

161. **Jouer à des jeux vidéos éducatifs (tablette, ordinateur, console...)** *

Une seule réponse possible.

- Ne s'applique pas à mon enfant
- Moins d'1 heure
- Entre 1 et 2 heures
- Entre 2 et 3 heures
- Entre 3 et 4 heures
- Plus de 4 heures

Sommeil

162. **En moyenne, combien d'heures par nuit votre enfant dort-il?** *

Une seule réponse possible.

- Moins de 6h
- Entre 6 et 7h
- Entre 7 et 8h
- Entre 8 et 9h
- Entre 9 et 10h
- Entre 10 et 11h
- Entre 11 et 12h
- Plus de 12h

Loisirs

163. **S'il vous plait, listez plusieurs activités que votre enfant aime faire pendant son temps libre :** *

S'il vous plait, listez trois des jeux et/ou jouets favoris de votre enfant

164. *

165. *

166. *

Livres

167. **S’il vous plait, estimez le nombre de livres pour enfants que vous possédez à votre domicile :** *

Une seule réponse possible.

- Aucun
- 1-10
- 11-20
- 21-40
- 41-60
- 61-80
- Autre :

168. **S'il vous plait, estimez le nombre de livres pour adultes que vous possédez à votre domicile :** *

Une seule réponse possible.

- Aucun
- 1-10
- 11-20
- 21-40
- 41-60
- 61-80
- Autre : ______

169. **Dans une semaine typique, combien de livres en moyenne lisez-vous à votre enfant? (si vous lisez deux fois le même livre, merci de compter "deux livres")** *

Une seule réponse possible.

- Jamais
- 1
- 2
- 3
- 4
- 5
- 6
- 7
- 8
- 9
- 10
- Plus de 10

170. **Voici une liste de livres adaptés aux enfants de 8 ans. Certains sont des livres réels, d'autres sont inventés. Cochez ceux qui vous sont familiers, que vous connaissez comme étant des titres de livres réels.** *

S'il vous plait, n'essayez pas de deviner, mais ne cochez que les titres de livres que vous connaissez.
Plusieurs réponses possibles.

- [ ] Le roman d'Ernest et Celestine
- [ ] L'os de Wily Fox
- [ ] Journal d'un chat assassin
- [ ] Autruche dans le désert
- [ ] Le lapin magique et autres histoires
- [ ] Chien pourri à l'école
- [ ] Le sage de la montagne fendue
- [ ] Qui a tué Minou-Bonbon ?
- [ ] La chanson de l'arbre au fond du jardin
- [ ] J'ai peur de savoir lire
- [ ] Enigmes à tous les étages
- [ ] Le chat de Tigali
- [ ] Mister M mène l'enquête
- [ ] La guerre des clans
- [ ] Journal d'un dégonflé
- [ ] A la croisée des mondes
- [ ] Grosse colère
- [ ] Les mots qui s'agitent

171. **S'il vous plait, écrivez ici les noms d'autres livres adaptés aux enfants de 8 ans que vous connaitriez :** *

Vous pouvez entrer les noms à la suite en les séparant par un espace ou une virgule (,)

Jeux

172. **S'il vous plait, estimez le nombre de jeux de société pour enfants que vous possédez à votre domicile :** *

Une seule réponse possible.

- Aucun
- 1-10
- 11-20
- 21-40
- 41-60
- 61-80
- Autre : ______________________

173. **S'il vous plait, estimez le nombre de jeux pour adultes que vous possédez à votre domicile :** *

Une seule réponse possible.

- Aucun
- 1-10
- 11-20
- 21-40
- 41-60
- 61-80
- Autre : ______________________

174. **Dans une semaine typique, combien de fois en moyenne jouez-vous à un jeu de société avec votre enfant?** *

Une seule réponse possible.

- Jamais
- 1
- 2
- 3
- 4
- 5
- 6
- 7
- 8
- 9
- 10
- Plus de 10

175. **Voici une liste de jeux adaptés aux enfants de 8 ans. Certains sont des jeux réels, d'autres sont inventés. Cochez ceux qui vous sont familiers, que vous connaissez comme étant des jeux réels.** *
S'il vous plait, n'essayez pas de deviner, mais ne cochez que les jeux que vous connaissez.
Plusieurs réponses possibles.

- [ ] La bonne paye
- [ ] Vocabulon
- [ ] Panic cafard
- [ ] Triominos
- [ ] Dobble 1,2,3
- [ ] Time's up!
- [ ] Course aux points
- [ ] Familou
- [ ] Bazar Bizarre
- [ ] Chomino
- [ ] Le jeu du prince de Mot tordu
- [ ] Rummikub
- [ ] Monopoly
- [ ] Tic tac boum junior
- [ ] Premier qui l'a dit!
- [ ] Tapo bac
- [ ] Tête de crotte
- [ ] Qui s'y frotte s'y piK

176. **S'il vous plait, écrivez ici les noms d'autres jeux de société adaptés aux enfants de 8 ans que vous connaitriez :** *
Vous pouvez entrer les noms à la suite en les séparant par un espace ou une virgule (,)

____
____
____
____
____

Durant le DERNIER MOIS, à quelle fréquence vous êtes-vous engagés dans les activités suivantes AVEC votre enfant ? Vous pouvez également cocher la case "Enfant seul" si votre enfant pratique cette activité mais sans votre participation. Si vous avez réalisé cette activité dans le passé mais ne la faites plus désormais, cocher "dans le

passé".

Description des activités réalisées dans le cadre familial

177. **Regarder des dessins animés, des séries ou films adaptés à son âge** *
Une seule réponse possible.

- N'a jamais lieu ou très rarement / L'activité ne s'applique pas à mon enfant.
- 1-3 fois par mois
- 1 fois par semaine
- 2-4 fois par semaine
- Quasiment tous les jours
- Quotidiennement
- Dans le passé
- Enfant seul

178. **Regarder des émissions de TV éducatives** *
Une seule réponse possible.

- N'a jamais lieu ou très rarement / L'activité ne s'applique pas à mon enfant.
- 1-3 fois par mois
- 1 fois par semaine
- 2-4 fois par semaine
- Quasiment tous les jours
- Quotidiennement
- Dans le passé
- Enfant seul

Durant le DERNIER MOIS, à quelle fréquence vous êtes-vous engagés dans les activités suivantes AVEC votre enfant ? Vous pouvez également cocher la case "Enfant seul" si votre enfant pratique cette activité mais sans votre participation. Si vous avez réalisé cette activité dans le passé mais ne la faites plus désormais, cocher "dans le passé".

Description des activités réalisées dans le cadre familial

179. **Se promener en ville/dans la nature** *

Une seule réponse possible.

- N’a jamais lieu ou très rarement / L'activité ne s'applique pas à mon enfant.
- 1-3 fois par mois
- 1 fois par semaine
- 2-4 fois par semaine
- Quasiment tous les jours
- Quotidiennement
- Dans le passé
- Enfant seul

180. **Aller au parc/à l'aire de jeux** *

Une seule réponse possible.

- N’a jamais lieu ou très rarement / L'activité ne s'applique pas à mon enfant.
- 1-3 fois par mois
- 1 fois par semaine
- 2-4 fois par semaine
- Quasiment tous les jours
- Quotidiennement
- Dans le passé
- Enfant seul

181. **Faire du vélo** *

Une seule réponse possible.

- N’a jamais lieu ou très rarement / L'activité ne s'applique pas à mon enfant.
- 1-3 fois par mois
- 1 fois par semaine
- 2-4 fois par semaine
- Quasiment tous les jours
- Quotidiennement
- Dans le passé
- Enfant seul

182. **Jouer au ballon** *

Une seule réponse possible.

- N’a jamais lieu ou très rarement / L'activité ne s'applique pas à mon enfant.
- 1-3 fois par mois
- 1 fois par semaine
- 2-4 fois par semaine
- Quasiment tous les jours
- Quotidiennement
- Dans le passé
- Enfant seul

Durant le DERNIER MOIS, à quelle fréquence vous êtes-vous engagés dans les activités suivantes AVEC votre enfant ? Vous pouvez également cocher la case "Enfant seul" si votre enfant pratique cette activité mais sans votre participation. Si vous avez réalisé cette activité dans le passé mais ne la faites plus désormais, cocher "dans le passé".

Description des activités réalisées dans le cadre familial

183. **Ecouter de la musique** *

Une seule réponse possible.

- N’a jamais lieu ou très rarement / L'activité ne s'applique pas à mon enfant.
- 1-3 fois par mois
- 1 fois par semaine
- 2-4 fois par semaine
- Quasiment tous les jours
- Quotidiennement
- Dans le passé
- Enfant seul

184. **Raconter des histoires inventées** *

Une seule réponse possible.

- N'a jamais lieu ou très rarement / L'activité ne s'applique pas à mon enfant.
- 1-3 fois par mois
- 1 fois par semaine
- 2-4 fois par semaine
- Quasiment tous les jours
- Quotidiennement
- Dans le passé
- Enfant seul

185. **Discuter de la journée d'école** *

Une seule réponse possible.

- N'a jamais lieu ou très rarement / L'activité ne s'applique pas à mon enfant.
- 1-3 fois par mois
- 1 fois par semaine
- 2-4 fois par semaine
- Quasiment tous les jours
- Quotidiennement
- Dans le passé
- Enfant seul

186. **Faire des courses/du shopping** *

Une seule réponse possible.

- N'a jamais lieu ou très rarement / L'activité ne s'applique pas à mon enfant. *Passez à la question 191.*
- 1-3 fois par mois
- 1 fois par semaine
- 2-4 fois par semaine
- Quasiment tous les jours
- Quotidiennement
- Dans le passé
- Enfant seul

Durant le DERNIER MOIS, à quelle fréquence vous êtes-vous engagés dans les activités suivantes AVEC votre enfant ? Vous pouvez également cocher la case "Enfant seul" si votre enfant pratique cette activité mais sans

votre participation. Si vous avez réalisé cette activité dans le passé mais ne la faites plus désormais, cocher "dans le passé".

Pendant les course partagées avec l'enfant

187. **Peser ou compter des achats dans les magasins (ex : 4 pommes ou 1kg de pommes de terre)** *

Une seule réponse possible.

- N'a jamais lieu ou très rarement / L'activité ne s'applique pas à mon enfant.
- 1-3 fois par mois
- 1 fois par semaine
- 2-4 fois par semaine
- Quasiment tous les jours
- Quotidiennement
- Dans le passé
- Enfant seul

188. **Compter de l'argent** *

Une seule réponse possible.

- N'a jamais lieu ou très rarement / L'activité ne s'applique pas à mon enfant.
- 1-3 fois par mois
- 1 fois par semaine
- 2-4 fois par semaine
- Quasiment tous les jours
- Quotidiennement
- Dans le passé
- Enfant seul

189. **Laisser l'enfant payer de petites courses au commerçant** *

Une seule réponse possible.

- N'a jamais lieu ou très rarement / L'activité ne s'applique pas à mon enfant.
- 1-3 fois par mois
- 1 fois par semaine
- 2-4 fois par semaine
- Quasiment tous les jours
- Quotidiennement
- Dans le passé
- Enfant seul

190. **Réaliser des comparaisons de grandeurs (ex : choisir la file d'attente la plus courte)** *

Une seule réponse possible.

- N'a jamais lieu ou très rarement / L'activité ne s'applique pas à mon enfant.
- 1-3 fois par mois
- 1 fois par semaine
- 2-4 fois par semaine
- Quasiment tous les jours
- Quotidiennement
- Dans le passé
- Enfant seul

Durant le DERNIER MOIS, à quelle fréquence vous êtes-vous engagés dans les activités suivantes AVEC votre enfant ? Vous pouvez également cocher la case "Enfant seul" si votre enfant pratique cette activité mais sans votre participation. Si vous avez réalisé cette activité dans le passé mais ne la faites plus désormais, cocher "dans le passé".

191. **Jouer** *

Une seule réponse possible.

- N'a jamais lieu ou très rarement / L'activité ne s'applique pas à mon enfant. *Passez à la question 205.*
- 1-3 fois par mois
- 1 fois par semaine
- 2-4 fois par semaine
- Quasiment tous les jours
- Quotidiennement
- Dans le passé
- Enfant seul

Durant le DERNIER MOIS, à quelle fréquence vous êtes-vous engagés dans les activités suivantes AVEC votre enfant ? Vous pouvez également cocher la case "Enfant seul" si votre enfant pratique cette activité mais sans votre participation. Si vous avez réalisé cette activité dans le passé mais ne la faites plus désormais, cocher "dans le passé".

Description des activités de jeu

192. **Jouer avec des puzzles** *

Une seule réponse possible.

- N'a jamais lieu ou très rarement / L'activité ne s'applique pas à mon enfant.
- 1-3 fois par mois
- 1 fois par semaine
- 2-4 fois par semaine
- Quasiment tous les jours
- Quotidiennement
- Dans le passé
- Enfant seul

193. **Jouer à la corde à sauter/à la marelle** *

Une seule réponse possible.

- N'a jamais lieu ou très rarement / L'activité ne s'applique pas à mon enfant.
- 1-3 fois par mois
- 1 fois par semaine
- 2-4 fois par semaine
- Quasiment tous les jours
- Quotidiennement
- Dans le passé
- Enfant seul

194. **Jouer avec des jeux de construction (type Legos technique, Mecano ou autres jeux similaires)** *

Une seule réponse possible.

- N'a jamais lieu ou très rarement / L'activité ne s'applique pas à mon enfant.
- 1-3 fois par mois
- 1 fois par semaine
- 2-4 fois par semaine
- Quasiment tous les jours
- Quotidiennement
- Dans le passé
- Enfant seul

195. **Réaliser des constructions avec des blocs** *

Une seule réponse possible.

- N’a jamais lieu ou très rarement / L'activité ne s'applique pas à mon enfant.
- 1-3 fois par mois
- 1 fois par semaine
- 2-4 fois par semaine
- Quasiment tous les jours
- Quotidiennement
- Dans le passé
- Enfant seul

196. **Jouer avec des poupées** *

Une seule réponse possible.

- N’a jamais lieu ou très rarement / L'activité ne s'applique pas à mon enfant.
- 1-3 fois par mois
- 1 fois par semaine
- 2-4 fois par semaine
- Quasiment tous les jours
- Quotidiennement
- Dans le passé
- Enfant seul

197. **Jouer avec de petites figurines** *

Une seule réponse possible.

- N’a jamais lieu ou très rarement / L'activité ne s'applique pas à mon enfant.
- 1-3 fois par mois
- 1 fois par semaine
- 2-4 fois par semaine
- Quasiment tous les jours
- Quotidiennement
- Dans le passé
- Enfant seul

Durant le DERNIER MOIS, à quelle fréquence vous êtes-vous engagés dans les activités suivantes AVEC votre enfant ? Vous pouvez également cocher la case "Enfant seul" si votre enfant pratique cette activité mais sans votre participation. Si vous avez réalisé cette activité dans le passé mais ne la faites plus désormais, cocher "dans le

passé".

Jeux de plateau/jeux de société

198. **Jouer à des jeux de plateau/jeux de société** *

Une seule réponse possible.

- N'a jamais lieu ou très rarement / L'activité ne s'applique pas à mon enfant. *Passez à la question 200.*
- 1-3 fois par mois
- 1 fois par semaine
- 2-4 fois par semaine
- Quasiment tous les jours
- Quotidiennement
- Dans le passé
- Enfant seul

Durant le DERNIER MOIS, à quelle fréquence vous êtes-vous engagés dans les activités suivantes AVEC votre enfant ? Vous pouvez également cocher la case "Enfant seul" si votre enfant pratique cette activité mais sans votre participation. Si vous avez réalisé cette activité dans le passé mais ne la faites plus désormais, cocher "dans le passé".

Jeux de plateau/jeux de société

199. **Jouer à des jeux de plateau/jeux de société avec un dé impliquant des nombres** *

Une seule réponse possible.

- N'a jamais lieu ou très rarement / L'activité ne s'applique pas à mon enfant.
- 1-3 fois par mois
- 1 fois par semaine
- 2-4 fois par semaine
- Quasiment tous les jours
- Quotidiennement
- Dans le passé
- Enfant seul

Durant le DERNIER MOIS, à quelle fréquence vous êtes-vous engagés dans les activités suivantes AVEC votre enfant ? Vous pouvez également cocher la case "Enfant seul" si votre enfant pratique cette activité mais sans votre participation. Si vous avez réalisé cette activité dans

le passé mais ne la faites plus désormais, cocher "dans le passé".

Jeux de carte

200. **Jouer à des jeux de cartes** *

Une seule réponse possible.

- N'a jamais lieu ou très rarement / L'activité ne s'applique pas à mon enfant. *Passez à la question 202.*
- 1-3 fois par mois
- 1 fois par semaine
- 2-4 fois par semaine
- Quasiment tous les jours
- Quotidiennement
- Dans le passé
- Enfant seul

Durant le DERNIER MOIS, à quelle fréquence vous êtes-vous engagés dans les activités suivantes AVEC votre enfant ? Vous pouvez également cocher la case "Enfant seul" si votre enfant pratique cette activité mais sans votre participation. Si vous avez réalisé cette activité dans le passé mais ne la faites plus désormais, cocher "dans le passé".

Jeux de carte

201. **Jouer à des jeux de cartes avec des nombres** *

Une seule réponse possible.

- N'a jamais lieu ou très rarement / L'activité ne s'applique pas à mon enfant.
- 1-3 fois par mois
- 1 fois par semaine
- 2-4 fois par semaine
- Quasiment tous les jours
- Quotidiennement
- Dans le passé
- Enfant seul

Durant le DERNIER MOIS, à quelle fréquence vous êtes-vous engagés dans les activités suivantes AVEC votre enfant ? Vous pouvez également cocher la case "Enfant seul" si votre enfant pratique cette activité mais sans

votre participation. Si vous avez réalisé cette activité dans le passé mais ne la faites plus désormais, cocher "dans le passé".

Jeux sur ordinateur/tablette

202. **Jouer à des jeux sur ordinateur/tablette** *

Une seule réponse possible.

- N'a jamais lieu ou très rarement / L'activité ne s'applique pas à mon enfant. *Passez à la question 205.*
- 1-3 fois par mois
- 1 fois par semaine
- 2-4 fois par semaine
- Quasiment tous les jours
- Quotidiennement
- Dans le passé
- Enfant seul

Durant le DERNIER MOIS, à quelle fréquence vous êtes-vous engagés dans les activités suivantes AVEC votre enfant ? Vous pouvez également cocher la case "Enfant seul" si votre enfant pratique cette activité mais sans votre participation. Si vous avez réalisé cette activité dans le passé mais ne la faites plus désormais, cocher "dans le passé".

Jeux sur ordinateur/tablette

203. **Jouer à des jeux sur ordinateur/tablette impliquant des nombres** *

Une seule réponse possible.

- N'a jamais lieu ou très rarement / L'activité ne s'applique pas à mon enfant.
- 1-3 fois par mois
- 1 fois par semaine
- 2-4 fois par semaine
- Quasiment tous les jours
- Quotidiennement
- Dans le passé
- Enfant seul

204. **Jouer à des jeux sur ordinateur/tablette impliquant de la lecture ou de l’orthographe** *

Une seule réponse possible.

- N’a jamais lieu ou très rarement / L'activité ne s'applique pas à mon enfant.
- 1-3 fois par mois
- 1 fois par semaine
- 2-4 fois par semaine
- Quasiment tous les jours
- Quotidiennement
- Dans le passé
- Enfant seul

Durant le DERNIER MOIS, à quelle fréquence vous êtes-vous engagés dans les activités suivantes AVEC votre enfant ? Vous pouvez également cocher la case "Enfant seul" si votre enfant pratique cette activité mais sans votre participation. Si vous avez réalisé cette activité dans le passé mais ne la faites plus désormais, cocher "dans le passé".

205. **Faire du bricolage/utiliser des outils** *

Une seule réponse possible.

- N’a jamais lieu ou très rarement / L'activité ne s'applique pas à mon enfant.
- 1-3 fois par mois
- 1 fois par semaine
- 2-4 fois par semaine
- Quasiment tous les jours
- Quotidiennement
- Dans le passé
- Enfant seul

206. **Aider à réparer des objets mécaniques (ex : vélo, jouets...)** *

Une seule réponse possible.

- N’a jamais lieu ou très rarement / L'activité ne s'applique pas à mon enfant.
- 1-3 fois par mois
- 1 fois par semaine
- 2-4 fois par semaine
- Quasiment tous les jours
- Quotidiennement
- Dans le passé
- Enfant seul

207. **Réaliser et trier des collections d'objets (ex : trier des vêtements par couleur, forme ou taille)** *

Une seule réponse possible.

- N’a jamais lieu ou très rarement / L'activité ne s'applique pas à mon enfant.
- 1-3 fois par mois
- 1 fois par semaine
- 2-4 fois par semaine
- Quasiment tous les jours
- Quotidiennement
- Dans le passé
- Enfant seul

208. **Mesurer des poids ou des distances** *

Une seule réponse possible.

- N’a jamais lieu ou très rarement / L'activité ne s'applique pas à mon enfant.
- 1-3 fois par mois
- 1 fois par semaine
- 2-4 fois par semaine
- Quasiment tous les jours
- Quotidiennement
- Dans le passé
- Enfant seul

209. **Discuter de température/de vitesse (ex : "il fait 5 degrés aujourd'hui, mets ton bonnet !")** *

Une seule réponse possible.

- N'a jamais lieu ou très rarement / L'activité ne s'applique pas à mon enfant.
- 1-3 fois par mois
- 1 fois par semaine
- 2-4 fois par semaine
- Quasiment tous les jours
- Quotidiennement
- Dans le passé
- Enfant seul

210. **"Chronométrer" la vitesse d'une activité (ex : compter jusqu'à 10 pour venir à table ; temps de brossage des dents)** *

Une seule réponse possible.

- N'a jamais lieu ou très rarement / L'activité ne s'applique pas à mon enfant.
- 1-3 fois par mois
- 1 fois par semaine
- 2-4 fois par semaine
- Quasiment tous les jours
- Quotidiennement
- Dans le passé
- Enfant seul

211. **Lire des textes présents autour de soi (ex : paquet de céréales, panneau de signalisation...)** *

Une seule réponse possible.

- N'a jamais lieu ou très rarement / L'activité ne s'applique pas à mon enfant.
- 1-3 fois par mois
- 1 fois par semaine
- 2-4 fois par semaine
- Quasiment tous les jours
- Quotidiennement
- Dans le passé
- Enfant seul

212. **Utiliser une calculatrice** *

Une seule réponse possible.

- N’a jamais lieu ou très rarement / L'activité ne s'applique pas à mon enfant.
- 1-3 fois par mois
- 1 fois par semaine
- 2-4 fois par semaine
- Quasiment tous les jours
- Quotidiennement
- Dans le passé
- Enfant seul

213. **Aller dans une bibliothèque (adaptée aux enfants)** *

Une seule réponse possible.

- N’a jamais lieu ou très rarement / L'activité ne s'applique pas à mon enfant.
- 1-3 fois par mois
- 1 fois par semaine
- 2-4 fois par semaine
- Quasiment tous les jours
- Quotidiennement
- Dans le passé
- Enfant seul

214. **Faire la cuisine et mesurer des ingrédients pour la recette** *

Une seule réponse possible.

- N’a jamais lieu ou très rarement / L'activité ne s'applique pas à mon enfant.
- 1-3 fois par mois
- 1 fois par semaine
- 2-4 fois par semaine
- Quasiment tous les jours
- Quotidiennement
- Dans le passé
- Enfant seul

215. **Utiliser des perles/fabriquer des bijoux** *

Une seule réponse possible.

- N’a jamais lieu ou très rarement / L'activité ne s'applique pas à mon enfant.
- 1-3 fois par mois
- 1 fois par semaine
- 2-4 fois par semaine
- Quasiment tous les jours
- Quotidiennement
- Dans le passé
- Enfant seul

Durant le DERNIER MOIS, à quelle fréquence vous êtes-vous engagés dans les activités suivantes AVEC votre enfant ? Vous pouvez également cocher la case "Enfant seul" si votre enfant pratique cette activité mais sans votre participation. Si vous avez réalisé cette activité dans le passé mais ne la faites plus désormais, cocher "dans le passé".

216. **Parler de l'heure (avec des montres/des horloges)** *

Une seule réponse possible.

- N’a jamais lieu ou très rarement / L'activité ne s'applique pas à mon enfant.
- 1-3 fois par mois
- 1 fois par semaine
- 2-4 fois par semaine
- Quasiment tous les jours
- Quotidiennement
- Dans le passé
- Enfant seul

217. **Parler de la date (avec un calendrier)** *

Une seule réponse possible.

- N’a jamais lieu ou très rarement / L'activité ne s'applique pas à mon enfant.
- 1-3 fois par mois
- 1 fois par semaine
- 2-4 fois par semaine
- Quasiment tous les jours
- Quotidiennement
- Dans le passé
- Enfant seul

218. **Utiliser des cartes/des plans** *

Une seule réponse possible.

- N’a jamais lieu ou très rarement / L'activité ne s'applique pas à mon enfant.
- 1-3 fois par mois
- 1 fois par semaine
- 2-4 fois par semaine
- Quasiment tous les jours
- Quotidiennement
- Dans le passé
- Enfant seul

219. **Discuter de directions vers des endroits familiers (ex : comment aller de l'école à la maison)** *

Une seule réponse possible.

- N’a jamais lieu ou très rarement / L'activité ne s'applique pas à mon enfant.
- 1-3 fois par mois
- 1 fois par semaine
- 2-4 fois par semaine
- Quasiment tous les jours
- Quotidiennement
- Dans le passé
- Enfant seul

220. **Composer des numéros de téléphone** *
Une seule réponse possible.

- N'a jamais lieu ou très rarement / L'activité ne s'applique pas à mon enfant.
- 1-3 fois par mois
- 1 fois par semaine
- 2-4 fois par semaine
- Quasiment tous les jours
- Quotidiennement
- Dans le passé
- Enfant seul

221. **Chanter des chansons** *
Une seule réponse possible.

- N'a jamais lieu ou très rarement / L'activité ne s'applique pas à mon enfant.
  Passez à la question 225.
- 1-3 fois par mois
- 1 fois par semaine
- 2-4 fois par semaine
- Quasiment tous les jours
- Quotidiennement
- Dans le passé
- Enfant seul

Durant le DERNIER MOIS, à quelle fréquence vous êtes-vous engagés dans les activités suivantes AVEC votre enfant ? Vous pouvez également cocher la case "Enfant seul" si votre enfant pratique cette activité mais sans votre participation. Si vous avez réalisé cette activité dans le passé mais ne la faites plus désormais, cocher "dans le passé".

Chansons

222. **Chanter des chansons sur l'alphabet** *
Une seule réponse possible.

- N'a jamais lieu ou très rarement / L'activité ne s'applique pas à mon enfant.
- 1-3 fois par mois
- 1 fois par semaine
- 2-4 fois par semaine
- Quasiment tous les jours
- Quotidiennement
- Dans le passé
- Enfant seul

223. **Chanter des chansons avec des nombres** *
Une seule réponse possible.

- N'a jamais lieu ou très rarement / L'activité ne s'applique pas à mon enfant.
- 1-3 fois par mois
- 1 fois par semaine
- 2-4 fois par semaine
- Quasiment tous les jours
- Quotidiennement
- Dans le passé
- Enfant seul

224. **Inventer des rimes** *
Une seule réponse possible.

- N'a jamais lieu ou très rarement / L'activité ne s'applique pas à mon enfant.
- 1-3 fois par mois
- 1 fois par semaine
- 2-4 fois par semaine
- Quasiment tous les jours
- Quotidiennement
- Dans le passé
- Enfant seul

Durant le DERNIER MOIS, à quelle fréquence vous êtes-vous engagés dans les activités suivantes AVEC votre enfant ? Vous pouvez également cocher la case "Enfant seul" si votre enfant pratique cette activité mais sans votre participation. Si vous avez réalisé cette activité dans

le passé mais ne la faites plus désormais, cocher "dans le passé".

225. **Compter des objets "visibles" dans la vie de tous les jours (ex : compter avec votre enfant combien il y de courriers dans la boite aux lettres)** *

Une seule réponse possible.

- N'a jamais lieu ou très rarement / L'activité ne s'applique pas à mon enfant.
- 1-3 fois par mois
- 1 fois par semaine
- 2-4 fois par semaine
- Quasiment tous les jours
- Quotidiennement
- Dans le passé
- Enfant seul

226. **Pratiquer des activités de comptage sans objet visible dans la vie de tous les jours (ex : faire réciter la "comptine numérique" : 1, 2, 3, 4, ...)** *

Une seule réponse possible.

- N'a jamais lieu ou très rarement / L'activité ne s'applique pas à mon enfant.
- 1-3 fois par mois
- 1 fois par semaine
- 2-4 fois par semaine
- Quasiment tous les jours
- Quotidiennement
- Dans le passé
- Enfant seul

227. **Entrainer à mémoriser des résultats d'addition (par exemple : 2+3)** *

Une seule réponse possible.

- N'a jamais lieu ou très rarement / L'activité ne s'applique pas à mon enfant.
- 1-3 fois par mois
- 1 fois par semaine
- 2-4 fois par semaine
- Quasiment tous les jours
- Quotidiennement
- Dans le passé
- Enfant seul

228. **Entrainer à mémoriser les tables (ex : tables de multiplication)** *
Une seule réponse possible.

- N'a jamais lieu ou très rarement / L'activité ne s'applique pas à mon enfant.
- 1-3 fois par mois
- 1 fois par semaine
- 2-4 fois par semaine
- Quasiment tous les jours
- Quotidiennement
- Dans le passé
- Enfant seul

229. **Verbaliser et comparer des quantités dans la vie de tous les jours (par ex: répondre à la question "combien y a-t-il de compotes dans le réfrigérateur?")** *
Une seule réponse possible.

- N'a jamais lieu ou très rarement / L'activité ne s'applique pas à mon enfant.
- 1-3 fois par mois
- 1 fois par semaine
- 2-4 fois par semaine
- Quasiment tous les jours
- Quotidiennement
- Dans le passé
- Enfant seul

230. **Encourager à réaliser des additions dans la vie de tous les jours (ex : "cela coute 25 euros et ceci coûte 13 euros, combien cela fait en tout?")** *
Une seule réponse possible.

- N'a jamais lieu ou très rarement / L'activité ne s'applique pas à mon enfant.
- 1-3 fois par mois
- 1 fois par semaine
- 2-4 fois par semaine
- Quasiment tous les jours
- Quotidiennement
- Dans le passé
- Enfant seul

Durant le DERNIER MOIS, à quelle fréquence vous êtes-vous engagés dans les activités suivantes AVEC votre enfant ? Vous pouvez également cocher la case "Enfant seul" si votre enfant pratique cette activité mais sans

votre participation. Si vous avez réalisé cette activité dans le passé mais ne la faites plus désormais, cocher "dans le passé".

231. **Encourager à réaliser des soustractions simples dans la vie de tous les jours (ex : 3-1)** *

Une seule réponse possible.

- N’a jamais lieu ou très rarement / L'activité ne s'applique pas à mon enfant. *Passez à la question 233.*
- 1-3 fois par mois
- 1 fois par semaine
- 2-4 fois par semaine
- Quasiment tous les jours
- Quotidiennement
- Dans le passé
- Enfant seul

Durant le DERNIER MOIS, à quelle fréquence vous êtes-vous engagés dans les activités suivantes AVEC votre enfant ? Vous pouvez également cocher la case "Enfant seul" si votre enfant pratique cette activité mais sans votre participation. Si vous avez réalisé cette activité dans le passé mais ne la faites plus désormais, cocher "dans le passé".

232. **Encourager à réaliser des soustractions complexes dans la vie de tous les jours (ex : 34-16)** *

Une seule réponse possible.

- N’a jamais lieu ou très rarement / L'activité ne s'applique pas à mon enfant.
- 1-3 fois par mois
- 1 fois par semaine
- 2-4 fois par semaine
- Quasiment tous les jours
- Quotidiennement
- Dans le passé
- Enfant seul

Durant le DERNIER MOIS, à quelle fréquence vous êtes-vous engagés dans les activités suivantes AVEC votre enfant ? Vous pouvez également cocher la case "Enfant

seul" si votre enfant pratique cette activité mais sans votre participation. Si vous avez réalisé cette activité dans le passé mais ne la faites plus désormais, cocher "dans le passé".

233. **Interroger sur des multiplications simples (ex : 2x3)** *

Une seule réponse possible.

- N'a jamais lieu ou très rarement / L'activité ne s'applique pas à mon enfant. *Passez à la question 235.*
- 1-3 fois par mois
- 1 fois par semaine
- 2-4 fois par semaine
- Quasiment tous les jours
- Quotidiennement
- Dans le passé
- Enfant seul

Durant le DERNIER MOIS, à quelle fréquence vous êtes-vous engagés dans les activités suivantes AVEC votre enfant ? Vous pouvez également cocher la case "Enfant seul" si votre enfant pratique cette activité mais sans votre participation. Si vous avez réalisé cette activité dans le passé mais ne la faites plus désormais, cocher "dans le passé".

234. **Interroger sur des multiplications complexes (ex : 12x6)** *

Une seule réponse possible.

- N'a jamais lieu ou très rarement / L'activité ne s'applique pas à mon enfant.
- 1-3 fois par mois
- 1 fois par semaine
- 2-4 fois par semaine
- Quasiment tous les jours
- Quotidiennement
- Dans le passé
- Enfant seul

Durant le DERNIER MOIS, à quelle fréquence vous êtes-vous engagés dans les activités suivantes AVEC votre enfant ? Vous pouvez également cocher la case "Enfant seul" si votre enfant pratique cette activité mais sans

votre participation. Si vous avez réalisé cette activité dans le passé mais ne la faites plus désormais, cocher "dans le passé".

235. **Discuter des notions de "partage", encourager à en réaliser (ex : partager des portions de nourriture en parts égales)** *

Une seule réponse possible.

- N'a jamais lieu ou très rarement / L'activité ne s'applique pas à mon enfant.
- 1-3 fois par mois
- 1 fois par semaine
- 2-4 fois par semaine
- Quasiment tous les jours
- Quotidiennement
- Dans le passé
- Enfant seul

236. **Interroger sur des divisions simples (ex : 6÷2)** *

Une seule réponse possible.

- N'a jamais lieu ou très rarement / L'activité ne s'applique pas à mon enfant. *Passez à la question 238.*
- 1-3 fois par mois
- 1 fois par semaine
- 2-4 fois par semaine
- Quasiment tous les jours
- Quotidiennement
- Dans le passé
- Enfant seul

Durant le DERNIER MOIS, à quelle fréquence vous êtes-vous engagés dans les activités suivantes AVEC votre enfant ? Vous pouvez également cocher la case "Enfant seul" si votre enfant pratique cette activité mais sans votre participation. Si vous avez réalisé cette activité dans le passé mais ne la faites plus désormais, cocher "dans le passé".

237. **Interroger sur des divisions plus complexes (60÷12)** *
Une seule réponse possible.

- N'a jamais lieu ou très rarement / L'activité ne s'applique pas à mon enfant.
- 1-3 fois par mois
- 1 fois par semaine
- 2-4 fois par semaine
- Quasiment tous les jours
- Quotidiennement
- Dans le passé
- Enfant seul

Durant le DERNIER MOIS, à quelle fréquence vous êtes-vous engagés dans les activités suivantes AVEC votre enfant ? Vous pouvez également cocher la case "Enfant seul" si votre enfant pratique cette activité mais sans votre participation. Si vous avez réalisé cette activité dans le passé mais ne la faites plus désormais, cocher "dans le passé".

Apprentissage de l'écriture numérique

238. **Apprendre à écrire les nombres jusqu'à 20** *
Une seule réponse possible.

- N'a jamais lieu ou très rarement / L'activité ne s'applique pas à mon enfant.
- 1-3 fois par mois
- 1 fois par semaine
- 2-4 fois par semaine
- Quasiment tous les jours
- Quotidiennement
- Dans le passé
- Enfant seul

Durant le DERNIER MOIS, à quelle fréquence vous êtes-vous engagés dans les activités suivantes AVEC votre enfant ? Vous pouvez également cocher la case "Enfant seul" si votre enfant pratique cette activité mais sans votre participation. Si vous avez réalisé cette activité dans le passé mais ne la faites plus désormais, cocher "dans le passé".

Apprentissage de l'écriture numérique

239. **Apprendre à écrire les nombres jusqu'à 100** *
Une seule réponse possible.

- N’a jamais lieu ou très rarement / L'activité ne s'applique pas à mon enfant. *Passez à la question 241.*
- 1-3 fois par mois
- 1 fois par semaine
- 2-4 fois par semaine
- Quasiment tous les jours
- Quotidiennement
- Dans le passé
- Enfant seul

Durant le DERNIER MOIS, à quelle fréquence vous êtes-vous engagés dans les activités suivantes AVEC votre enfant ? Vous pouvez également cocher la case "Enfant seul" si votre enfant pratique cette activité mais sans votre participation. Si vous avez réalisé cette activité dans le passé mais ne la faites plus désormais, cocher "dans le passé".

Apprentissage de l'écriture numérique

240. **Apprendre à écrire les nombres jusqu'à 1000** *
Une seule réponse possible.

- N’a jamais lieu ou très rarement / L'activité ne s'applique pas à mon enfant. *Passez à la question 242.*
- 1-3 fois par mois
- 1 fois par semaine
- 2-4 fois par semaine
- Quasiment tous les jours
- Quotidiennement
- Dans le passé
- Enfant seul

Durant le DERNIER MOIS, à quelle fréquence vous êtes-vous engagés dans les activités suivantes AVEC votre enfant ? Vous pouvez également cocher la case "Enfant seul" si votre enfant pratique cette activité mais sans votre participation. Si vous avez réalisé cette activité dans le passé mais ne la faites plus désormais, cocher "dans le passé".

Apprentissage de l'écriture numérique

241. **Apprendre à lire les nombres jusqu'à 20** *
Une seule réponse possible.

- N'a jamais lieu ou très rarement / L'activité ne s'applique pas à mon enfant.
- 1-3 fois par mois
- 1 fois par semaine
- 2-4 fois par semaine
- Quasiment tous les jours
- Quotidiennement
- Dans le passé
- Enfant seul

Durant le DERNIER MOIS, à quelle fréquence vous êtes-vous engagés dans les activités suivantes AVEC votre enfant ? Vous pouvez également cocher la case "Enfant seul" si votre enfant pratique cette activité mais sans votre participation. Si vous avez réalisé cette activité dans le passé mais ne la faites plus désormais, cocher "dans le passé".

Apprentissage de l'écriture numérique

242. **Apprendre à lire les nombres jusqu'à 100** *
Une seule réponse possible.

- N'a jamais lieu ou très rarement / L'activité ne s'applique pas à mon enfant.
  Passez à la question 244.
- 1-3 fois par mois
- 1 fois par semaine
- 2-4 fois par semaine
- Quasiment tous les jours
- Quotidiennement
- Dans le passé
- Enfant seul

Durant le DERNIER MOIS, à quelle fréquence vous êtes-vous engagés dans les activités suivantes AVEC votre enfant ? Vous pouvez également cocher la case "Enfant seul" si votre enfant pratique cette activité mais sans votre participation. Si vous avez réalisé cette activité dans le passé mais ne la faites plus désormais, cocher "dans le passé".

Apprentissage de l'écriture numérique

243. **Apprendre à lire les nombres jusqu'à 1000** *

Une seule réponse possible.

- N’a jamais lieu ou très rarement / L'activité ne s'applique pas à mon enfant.
- 1-3 fois par mois
- 1 fois par semaine
- 2-4 fois par semaine
- Quasiment tous les jours
- Quotidiennement
- Dans le passé
- Enfant seul

Durant le DERNIER MOIS, à quelle fréquence vous êtes-vous engagés dans les activités suivantes AVEC votre enfant ? Vous pouvez également cocher la case "Enfant seul" si votre enfant pratique cette activité mais sans votre participation. Si vous avez réalisé cette activité dans le passé mais ne la faites plus désormais, cocher "dans le passé".

Pratique de l'écriture

244. **Encourager la rédaction de textes courts (ex : journal intime)** *

Une seule réponse possible.

- N’a jamais lieu ou très rarement / L'activité ne s'applique pas à mon enfant. *Passez à la question 246.*
- 1-3 fois par mois
- 1 fois par semaine
- 2-4 fois par semaine
- Quasiment tous les jours
- Quotidiennement
- Dans le passé
- Enfant seul

Durant le DERNIER MOIS, à quelle fréquence vous êtes-vous engagés dans les activités suivantes AVEC votre enfant ? Vous pouvez également cocher la case "Enfant seul" si votre enfant pratique cette activité mais sans votre participation. Si vous avez réalisé cette activité dans le passé mais ne la faites plus désormais, cocher "dans le passé".

Pratique de l'écriture

245. **Encourager la rédaction de textes longs (ex : histoire, lettres ou courrier)** *
Une seule réponse possible.

- N'a jamais lieu ou très rarement / L'activité ne s'applique pas à mon enfant.
- 1-3 fois par mois
- 1 fois par semaine
- 2-4 fois par semaine
- Quasiment tous les jours
- Quotidiennement
- Dans le passé
- Enfant seul

Durant le DERNIER MOIS, à quelle fréquence vous êtes-vous engagés dans les activités suivantes AVEC votre enfant ? Vous pouvez également cocher la case "Enfant seul" si votre enfant pratique cette activité mais sans votre participation. Si vous avez réalisé cette activité dans le passé mais ne la faites plus désormais, cocher "dans le passé".

Pratique de la lecture

246. **Encourager la lecture de textes courts** *
Une seule réponse possible.

- N'a jamais lieu ou très rarement / L'activité ne s'applique pas à mon enfant. *Passez à la question 248.*
- 1-3 fois par mois
- 1 fois par semaine
- 2-4 fois par semaine
- Quasiment tous les jours
- Quotidiennement
- Dans le passé
- Enfant seul

Durant le DERNIER MOIS, à quelle fréquence vous êtes-vous engagés dans les activités suivantes AVEC votre enfant ? Vous pouvez également cocher la case "Enfant seul" si votre enfant pratique cette activité mais sans votre participation. Si vous avez réalisé cette activité dans le passé mais ne la faites plus désormais, cocher "dans le passé".

Pratique de la lecture

247. **Encourager la lecture de chapitres entiers de livre** *

Une seule réponse possible.

- N’a jamais lieu ou très rarement / L'activité ne s'applique pas à mon enfant.
- 1-3 fois par mois
- 1 fois par semaine
- 2-4 fois par semaine
- Quasiment tous les jours
- Quotidiennement
- Dans le passé
- Enfant seul

Durant le DERNIER MOIS, à quelle fréquence vous êtes-vous engagés dans les activités suivantes AVEC votre enfant ? Vous pouvez également cocher la case "Enfant seul" si votre enfant pratique cette activité mais sans votre participation. Si vous avez réalisé cette activité dans le passé mais ne la faites plus désormais, cocher "dans le passé".

Pratique de la lecture

248. **Ecouter lorsque votre enfant lit à haute voix** *

Une seule réponse possible.

- N’a jamais lieu ou très rarement / L'activité ne s'applique pas à mon enfant.
- 1-3 fois par mois
- 1 fois par semaine
- 2-4 fois par semaine
- Quasiment tous les jours
- Quotidiennement
- Dans le passé

249. **Poser des questions sur ses lectures** *

Une seule réponse possible.

- N’a jamais lieu ou très rarement / L'activité ne s'applique pas à mon enfant.
- 1-3 fois par mois
- 1 fois par semaine
- 2-4 fois par semaine
- Quasiment tous les jours
- Quotidiennement
- Dans le passé

Durant le DERNIER MOIS, à quelle fréquence vous êtes-vous engagés dans les activités suivantes AVEC votre enfant ? Vous pouvez également cocher la case "Enfant seul" si votre enfant pratique cette activité mais sans votre participation. Si vous avez réalisé cette activité dans le passé mais ne la faites plus désormais, cocher "dans le passé".

Pratique de la lecture

250. **Lire ensemble** *

Une seule réponse possible.

- N'a jamais lieu ou très rarement / L'activité ne s'applique pas à mon enfant.
- 1-3 fois par mois
- 1 fois par semaine
- 2-4 fois par semaine
- Quasiment tous les jours
- Quotidiennement
- Dans le passé

251. **Interroger l'enfant lors d'une lecture partagée** *

Une seule réponse possible.

- N'a jamais lieu ou très rarement / L'activité ne s'applique pas à mon enfant.
- 1-3 fois par mois
- 1 fois par semaine
- 2-4 fois par semaine
- Quasiment tous les jours
- Quotidiennement
- Dans le passé

Durant le DERNIER MOIS, à quelle fréquence vous êtes-vous engagés dans les activités suivantes AVEC votre enfant ? Vous pouvez également cocher la case "Enfant seul" si votre enfant pratique cette activité mais sans votre participation. Si vous avez réalisé cette activité dans le passé mais ne la faites plus désormais, cocher "dans le passé".

252. **Apprendre et corriger l'orthographe** *

Une seule réponse possible.

- N'a jamais lieu ou très rarement / L'activité ne s'applique pas à mon enfant.
- 1-3 fois par mois
- 1 fois par semaine
- 2-4 fois par semaine
- Quasiment tous les jours
- Quotidiennement
- Dans le passé
- Enfant seul

253. **Interroger sur la conjugaison des verbes** *

Une seule réponse possible.

- N'a jamais lieu ou très rarement / L'activité ne s'applique pas à mon enfant.
- 1-3 fois par mois
- 1 fois par semaine
- 2-4 fois par semaine
- Quasiment tous les jours
- Quotidiennement
- Dans le passé
- Enfant seul

Merci pour votre participation !

Fourni par Google Forms
