# Supplementary material for: Prior home learning environment is associated with adaptation to homeschooling during COVID lockdown
Source: Heliyon. 2022 Apr 19;8(4):e09294. doi: 10.1016/j.heliyon.2022.e09294 (PMC9017091; doi:10.1016/j.heliyon.2022.e09294)
Supplement: Parental-questionnaire-T1-English-translation.pdf [file mmc5.pdf]

Parental Questionnaire T1 (English translation)

• Are you the mother or the father of the child in the study?

• What is your postal code?

• What is your year of birth?

• What is your place of birth?

• What is the highest level of education you have completed?
XXX

• Which of these categories best describes your current work situation? *I am a student or in training; I have a full-time job; I have a part-time job; I am currently unemployed*

• Which of these categories describes most accurately your occupation? *Farmer; Craftsman, merchant and company manager; Managers and professionals in higher education Intermediate professions; Employee; Worker; Inactive (never worked); Other*

• What is your net monthly income? *0 € - 999 €;1,000 € - 1,999 €; 2,000 € - 2,999 €; 3,000 € - 3,999 €; 4,000 € - 4,999 €; 5,000 € - 5,999 €;6,000 € - 6,999 €;7,000 € - 7,999 €; 8,000 € - 8,999 €; 9,000 € - 9,999 €; 10,000 € and more*

• What languages are spoken at home?

• Have you ever been diagnosed with a learning disability (a disability that affects YOU)? *Yes; No*

If yes : . *Which one(s)? Dsylexia (learning to read); Dyscalculia (learning to do math); Dyspraxia (motor development and writing disorder); Dysphasia (oral language disorder); ADD/ADHD (attention deficit disorder)*
And at what age were you diagnosed?

• Has anyone else in your family (other than the child in the study) ever been diagnosed with a learning disability?)? *Yes; No*

If yes : . *Which one(s)? Dsylexia (learning to read); Dyscalculia (learning to do math); Dyspraxia (motor development and writing disorder); Dysphasia (oral language disorder); ADD/ADHD (attention deficit disorder)*
And at what age was s.he diagnosed?

• Read the following statements. Please indicate how much you agree with the statements using the scale provided by checking the appropriate box.

	Don't know	Strongly Disagree	Disagree	Agree	Strongly Agree
When I was in school, I was good at math.					
When I was in school, I was good at language-related					

activities, like reading.					
My job involves a lot of written reports.					
My job involves using math.					
I find math enjoyable.					
I find writing enjoyable.					
I find reading enjoyable.					
I avoid situations involving writing					
I avoid situations involving reading					
I avoid situations involving math					

• Currently, in addition to yourself, how many adults live in your home?

• Currently, in addition to yourself, how many children live in your home?

• Read the following statements. Please indicate how much you agree with the statements using the scale provided by checking the appropriate box.

	Don't know	Strongly Disagree	Disagree	Agree	Strongly Agree
I am confident that I know how to involve my child in a math activity.					
I am confident that I know how to involve my child in a reading activity.					
In my home, math skills are taught randomly throughout the day.					
In my home, reading skills are taught randomly throughout the day.					

• How important do you think it is for your child to acquire the following benchmarks before the end of elementary school?

	No opinion	Not at all important	Not important	Important	Very important	Extremely important
Know how to communicate with adults (outside the family).						
Develop and maintain friendships with children of the same age.						
Apply instructions and respect the rules of collective life.						

Know and locate major geographical landmarks on various maps.						
Identify some of the challenges of durable development.						
Know and situate major historical periods in time.						
Count to 100.						
Count to 1000.						
Read written numbers to 100 (1, 2, 3, ...).						
Read written numbers up to 1000 (1, 2, 3, ...).						
Make simple sums without counting on one's fingers (e.g. 2+2).						
Know how to do complex additions (e.g. 15+121; 11+447).						
Know how to do simple multiplication (ex : 2x6).						
Know how to do more complex multiplications (ex : 14x7).						
Know fractions and how to use them (ex : 5/6).						
Know how to do divisions (ex : 30÷5).						
Know the decimal numbers (ex : 3,2).						
Calculate with decimal numbers.						
Know and use probabilities.						
Recognize, name and reproduce geometric shapes.						
Recognize and use some geometric concepts (e.g., perpendicularity and parallelism).						
Know how to use a ruler to measure a length.						
Express a measured or calculated quantity in an appropriate unit.						
Write by hand in a fluent and efficient manner.						
Write a text of about half a page.						
Write independently (e.g., to keep track of what they read).						
Know how to spell frequent words.						
Know how to spell memorized invariable words (e.g., then, in, finally...).						
Read simple picture books (=books with pictures and text).						
Read and understand short paragraphs.						
Read chapter books (books with text only).						
Read an entire children's book.						
Understand and interpret texts or works.						

Make an oral presentation, an activity report (e.g., presentation).						
Participate in oral exchanges (e.g., debate).						
Implement an experimental protocol, design or produce all or part of a technical object.						
Practice various forms of literary and artistic creation.						
Perform sports (running, jumping, etc.).						
Understand the general functioning of the human body.						

• How would you rate your child's abilities in the following domains?

	No opinion	Major difficulties	Average ability	Very good abilities	Extremely good abilities
Writing					
Spelling					
Reading					
Mathematics					
Science					
History / Geography					
Music					
Arts					
Sports					

• How important is your child’s academic success in these different domains?

	No opinion	Not at all important	Not important	Important	Very important	Extremely important
Writing						
Spelling						
Reading						
Mathematics						
Science						
History / Geography						
Music						
Arts						
Sports						

• How often do you ask your child to do exercises/activities in the following domains (outside of school homework)?

	Never	Less than 15 minutes per day	About 15-30 minutes per day	About 30 minutes -1h	More than 1 hour per day
Mathematics (e.g., mental math).					
Reading (e.g., reading books silently or aloud).					

Spelling (e.g., dictating words).					
Writing (e.g., writing letters, essays; taking notes).					
Science (e.g., planting seeds).					
Music (e.g., playing an instrument).					
Art (e.g., making paintings).					
Sports (e.g., playing a sport).					

• Did your child attend preschool between the ages of 2 and 3? *Yes; No*
If yes : On average, how many hours per week did your child attend preschool between the ages of 2 and 3?

• In a typical week (excluding weekends), how many hours per day on average do you spend with your child? *Less than 1 hour; Between 1 and 2 hours; Between 2 and 3 hours; Between 3 and 4 hours; Between 4 and 5 hours; Between 5 and 6 hours; Between 6 and 7 hours; Over 7 hours.*

• In a typical week, on average, how many hours per day does your child spend doing the following activities? *Not applicable to my child*; *Less than 1 hour; Between 1 and 2 hours; Between 2 and 3 hours; Between 3 and 4 hours; more than 4 hours*

- watching non-educational cartoons or TV shows
- watching educational TV programs
- playing non-educational video games (tablet, computer, console...)
- playing educational video games (tablet, computer, console...)

• On average, how many hours per night does your child sleep? *Less than 6 hours; Between 6 and 7 hours; Between 7 and 8 hours; Between 8 and 9 hours; Between 9 and 10 hours; Between 10 and 11 hours; Between 11 and 12 hours; Over 12 hours*

• List several activities that your child likes to do in his/her free time:

• List three of your child's favorite games and/or toys
• Estimate how many children's books you have in your home: *None; 1-10; 11-20; 21-40; 41-60; 61-80; Other*

• Estimate how many adult books you have in your home: *None; 1-10; 11-20; 21-40; 41-60; 61-80; Other*

• In a typical week, how many books on average do you read to your child? (if you read the same book twice, please count "two books") *Never; 1; 2; 3; 4; 5; 6; 7; 8; 9; 10; More than 10.*

• Here is a list of books suitable for 8 year olds. Some are real books, some are made up. Check off the ones you are familiar with, that you know are real book titles.

• Please write the names of other books suitable for 8 year olds that you know of.

• Estimate how many children's board games you have in your home: *None; 1-10; 11-20; 21-40; 41-60; 61-80; Other.*

• Estimate how many adult's board games you have in your home: *None; 1-10; 11-20; 21-40; 41-60; 61-80; Other.*

• In a typical week, how many times on average do you play a board game with your child? *Never; 1; 2; 3; 4; 5; 6; 7; 8; 9; 10; More than 10.*

• Here is a list of board games suitable for 8 year olds. Some are real board games; some are made up. Check off the ones you are familiar with, that you know are real board games names.

• Please write the names of other board games suitable for 8 year olds that you know of.

• During the LAST MONTH, how often did you engage in the following activities WITH your child?

	Never happens or very rarely / The activity does not apply to my child.	1-3 times a month	1 time per week	2-4 times a week	Almost daily	Daily	In the past	Child alone
Watching cartoons, series or movies								
Watching educational TV programs								
Going for walks in the city/nature								
Going to the park/playground								
Riding a bike								
Playing ball								
Listening to music								
Telling invented stories								
Discussing the school day								
Shopping								
Weighing or counting purchases in stores (e.g., 4 apples or 1 kg of potatoes)								
Counting money								
Letting the child pay for small errands at the store								
Making size comparisons (e.g., choosing the shortest line)								
Play								

Playing with puzzles								
Playing jump rope/hopscotch								
Playing with construction games (e.g., Lego)								
Building with blocks								
Playing with dolls								
Playing with small figurines								
Playing board games								
Playing board games with a dice involving numbers								
Playing card games								
Playing card games with numbers								
Playing computer/tablet games								
Play computer/tablet games involving numbers								
Playing computer/tablet games involving reading or spelling								
Doing crafts/using tools								
Helping to repair mechanical objects (e.g., bicycle, toys, etc.)								
Making and sorting collections of objects (e.g., sorting clothes by color, shape or size)								
Measure weights or distances								
Discuss temperature/speed (e.g., "It's 5 degrees today, put on your hat!")								
"Timing" the speed of an activity (e.g., counting to 10 to come to the table; time to brush teeth)								

Read texts around you (e.g., cereal packets, road signs, etc.)								
Use a calculator								
Go to children library								
Cooking and measuring ingredients for a recipe								
Use beads/jewelry making								
Talking about time (with watches/clocks)								
Talking about the date (with a calendar)								
Using maps/plans								
Discussing directions to familiar places (e.g., how to get from school to home)								
Dialing phone numbers								
Singing songs								
Singing songs about the alphabet								
Singing songs with numbers								
Making up rhymes								
Counting "visible" objects in everyday life (e.g., counting with your child how many letters are in the mailbox)								
Practice counting activities without visible objects in everyday life (e.g., have your child recite the "number count": 1, 2, 3, 4, ...)								
Practice memorizing addition results (e.g., 2+3)								
Practice memorizing tables (e.g., multiplication tables)								
Verbalize and compare quantities in everyday life (e.g., answer the question "how many compotes are in the refrigerator?")								

Encourage addition in everyday life (e.g., "this costs 25 euros and this costs 13 euros, how much is that in total?")								
Encourage simple subtraction in everyday life (e.g., 3-1)								
Encourage complex subtractions in everyday life (e.g. 34-16)								
Ask about simple multiplications (e.g. 2x3)								
Ask about more complex multiplications (e.g., 12x6)								
Discuss and encourage "sharing" (e.g., sharing portions of food equally)								
Ask about simple division (e.g., 6÷2)								
Ask about more complex divisions (60÷12)								
Learn to write numbers up to 20								
Learn to write numbers to 100								
Learn to write numbers up to 1000								
Learn to read numbers to 20								
Learn to read numbers to 100								
Learn to read numbers up to 1000								
Encourage writing of short texts (e.g., diary)								
Encourage writing of longer texts (e.g., stories, letters or letters)								
Encourage reading of short texts								
Encourage reading of entire chapters of books								
Listen when your child reads aloud								

Ask questions about what he or she is reading								
Read together								
Question the child when reading together								
Learn and correct spelling								
Ask about verb conjugation								
